# Sodium in Canadian processed foods between 2010 and 2020: implications for future sodium reduction initiatives

**DOI:** 10.1017/S1368980026102067

**Published:** 2026-02-20

**Authors:** Emily R. Ziraldo, Yahan Yang, JoAnne Arcand, Anthea Christoforou, Jennifer J. Lee, Mary R. L’Abbé

**Affiliations:** 1 Department of Nutritional Sciences, Temerty Faculty of Medicine, University of Toronto, Toronto, ON, Canada; 2 Faculty of Health Sciences, Ontario Tech University, Oshawa, ON, Canada; 3 Department of Kinesiology, Faculty of Sciences, McMaster University, Hamilton, ON, Canada (current address for A.C.); 4 School of Nutrition, Faculty of Community Services, Toronto Metropolitan University, Toronto, ON, Canada (current address for J.J.L); 5 Joannah & Brian Lawson Centre for Child Nutrition, Temerty Faculty of Medicine, University of Toronto, Toronto, ON, Canada

**Keywords:** Food supply, Packaged foods, Processed foods, Na reduction, Nutrition policy

## Abstract

**Objectives:**

To assess changes in (1) Na content of processed foods in the Canadian food supply and (2) the proportion of products meeting Health Canada’s voluntary Na reduction targets (SRT) between 2010 and 2020.

**Design:**

This repeated, cross-sectional study used foods from the 2010 (*n* 6929), 2013 (*n* 9366), 2017 (*n* 10 324) and 2020 (*n* 15 797) collections of the University of Toronto’s Food Label Information and Price database, categorised into Health Canada’s Na categories. Quantile regression was used to assess changes in Na content. Firth’s bias-reduced logistic regression was used to evaluate changes in the proportion of foods meeting the SRT, and trends were assessed with Cochran–Armitage tests.

**Setting:**

Canada.

**Participants:**

Processed foods.

**Results:**

Between 2010 and 2020, 54 % (7/13) of major categories had a left shift (reduction) in their Na distribution, 15 % (2/13) had a right shift (increase), 15 % (2/13) had both a left and right shift and 15 % (2/13) did not change. The proportion of products meeting the average targets and maximum levels increased 6 % and 4 % from 2010 to 2013 and 4 % and 3 % from 2013 to 2017, then decreased 3 % and 1 % between 2017 and 2020, with trends for improvement over time (*P*-trend < 0·001).

**Conclusions:**

Although many categories decreased in Na, some did not change or increased in Na and improvements in the proportion of products meeting the SRT were modest and occurred early on. Further actions, such as implementing accountability initiatives that promote industry adherence to voluntary SRT or introducing mandatory measures, alongside frequent and transparent monitoring are needed to reduce Na in processed foods in Canada.

Globally, CVD is the leading cause of death and disability^(1)^. Excess dietary Na intake raises blood pressure and, consequently, increases risk for CVD^(2)^. Reducing Na intakes can lower blood pressure and decrease CVD risk^(2,3)^. In 2010, 96·8 % of countries exceeded the WHO recommended intake level of 2000 mg/d^(4)^. The WHO Global Action Plan for the Prevention and Control of Non-Communicable Diseases (NCD-GAP 2013–2020), which was extended to 2030 (NCD-GAP 2013–2030), identified a 30 % relative reduction in mean population Na intakes as one of nine voluntary global targets to reduce the burden of NCD^(5)^.

In Canada, CVD has been the second leading cause of death for over 20 years,^(6)^ and Health Canada has identified Na reduction as a national public health priority^(7)^. Canadians consume an average of 2760 mg of Na per day, exceeding the recommended limit of 2300 mg and over 58 % of Canadians ≥ 1 year old exceed their age- and sex-specific guidelines^(8)^. The top food categories contributing to Canadians’ Na intake are bakery products, mixed dishes, processed meats, cheeses and soups^(8)^, with the majority of Na intakes (75–77 %) coming from Na adding during manufacturing and processing^(9,10)^. Since most Na consumed in Canada is already present in foods at purchase, reducing Na in the food supply is likely more effective at reducing intakes than targeting Na added by consumers.

In 2007, the Minister for Health announced the creation of a multi-stakeholder Sodium Working Group to develop a strategy for reducing Canadians’ Na intakes. In 2010, the Sodium Working Group published the Sodium Reduction Strategy for Canada with strategic recommendations to reduce Na consumption^(11)^. One recommendation was a structured voluntary approach to Na reduction, which included establishing Na reduction targets (SRT) for foods with defined timelines, providing a mechanism for industry to publicly commit to meeting the SRT and implementing both a monitoring plan to track progress and an evaluation plan, with the possibility of stronger measures if progress was insufficient^(11)^. However, the Sodium Working Group was disbanded in early 2011, prior to completing the implementation and monitoring components of the Group’s Terms of Reference.

In 2012, Health Canada published voluntary SRT to encourage food manufacturers to incrementally lower Na levels in processed foods, with the aim of a 25–30 % reduction in sales-weighted average Na content across most food categories by 2016^(12)^. The SRT include two types of goal targets: (1) a sales-weighted average target that manufacturers should aim for the average sales-weighted Na content of their portfolio to meet and (2) a maximum level, which no products should exceed. In 2020, the voluntary SRT were updated with new targets to be achieved by 2025, although the majority did not change^(13)^. The updates included splitting or adding target categories and changes to targets to make them more realistic or to maintain progress within a category^(13)^. Lowering the Na content of processed foods, also known as reformulation, and setting SRT for foods are recommended by the WHO as one of 16 ‘best buys’, which are impactful, cost-effective and feasible interventions that can help achieve one of the global targets in NCD-GAP 2013–2030^(14)^. However, in the 2023 WHO Global Report on Na Intake Reduction, the WHO recommended mandatory, rather than voluntary, approaches to Na reduction^(1)^.

There have been three previous comprehensive assessments of progress towards Na reduction in processed foods in Canada and one interim assessment of select food subcategories. The first assessed progress between 2010 and 2013 using the Food Label Information and Price (FLIP) database of Canadian processed foods, finding most categories (81·9 %) did not change in Na levels and the proportion of foods meeting the average targets increased slightly, from 28·6 % in 2010 to 33·6 % in 2013^(15)^. Health Canada published an evaluation of the SRT in 2017 using a sample of foods from each category that were selected based on sales data, reporting that almost half (48 %) of categories had not made progress towards reducing Na and only 14 % met the average targets^(16)^. An updated analysis of the first study assessed progress between 2013 and 2017 using the FLIP database and found that 59 % of categories had no change in Na content and the proportion of foods meeting the average targets increased marginally, from 33·6 % in 2013 to 37·3 % in 2017^(17)^. Lastly, Health Canada released an interim assessment of Na reduction in nine select food subcategories between 2017 and 2023/2024, finding that five categories made progress towards their sales-weighted average targets and four increased in Na^(18)^. All four assessments showed that progress towards Na reduction has been limited and suggest that stronger initiatives are needed to make substantial progress.

Despite Na reduction remaining a priority for the federal government,^(7)^ no comprehensive long-term assessment of Na levels in the Canadian processed food supply has been published using data collected after 2017. Considering the potential lag in reformulation, release of new voluntary SRT in 2020 and the lack of centralised data from previous assessments, an updated and longer-term evaluation is needed to summarise the impact of the first voluntary SRT and identify opportunities for further action. Leveraging the longest running food composition database of Canadian foods, FLIP, we assessed changes in Na content of processed foods sold in Canada between 2010 and 2020, using cross-sectional data from four timepoints: 2010, 2013, 2017 and 2020. The secondary aim was to evaluate changes in the proportion of products meeting Health Canada’s 2012–2016 voluntary SRT at each time point.

## Methods

This study was a repeated, cross-sectional examination of Na content in processed foods, collected in 2010, 2013, 2017 and 2020.

### Data collection and preparation

Data were obtained from the University of Toronto’s Food Label Information and Price (FLIP) database, with details described elsewhere^(19–22)^. Briefly, FLIP is a branded food composition database including foods and beverages from stores representing 56–80 % of Canada’s grocery retail market share in 2010, 2013, 2017 and 2020. Data for FLIP 2010, 2013 and 2017 were collected by going into three or four top grocery retailers by market share and systematically taking photos of label information for packaged foods and beverages. Data for the FLIP 2020 collection were collected by web scraping seven top grocery retailer websites for label information for all foods and beverages available online. Label data in FLIP include Na levels and serving size as written on the Nutrition Facts table (NFt), the product name, ingredients list (only FLIP 2013, 2017 and 2020) and images of the product packaging (as available on websites in FLIP 2020). Products were categorised into the Na category hierarchy, which includes major (e.g. bakery products), sub (e.g. bread products and cookies) and minor (e.g. pantry bread, hearth bread, rolls and buns) categories as established by Health Canada^(12)^. In alignment with previous assessments, some subcategories were grouped together (e.g. canned vegetables and legumes, sour picked vegetables, olives and sundried tomatoes, stuffed olives, vegetable juice and cocktails were grouped as canned vegetables and legumes)^(15,17)^. Products that did not have a corresponding Na category (e.g. juice, dried pasta or rice without seasonings) and those in categories without SRT (e.g. unsalted butter) were excluded, as these products are generally low in Na or do not contribute significantly to Canadians’ Na intakes^(12)^.

Na levels in mg/100 g or mg/100 ml were determined using the Na level (mg/serving) and serving size (g or ml) on the NFt. To express all Na levels in mg/100 g, products with Na levels in mg/100 ml, were converted to mg/100 g using millilitre to grams conversion factors from the Canadian Nutrient File, a reference food composition database developed by the Government of Canada^(23)^. ‘As prepared’ Na values for products that required preparation (e.g. soup concentrate and pancake mixes) were calculated according to the recipes available on the package. When no specific recipe was available or when products had similar recipes, a general recipe for a food category was used. The Canadian Nutrient File was used to obtain reference nutrient values of the additional ingredients (e.g. milk added to dry pancake mixes)^(23)^. For categories with SRT for unprepared or ‘as sold’ products (e.g. hot instant cereals), the unprepared Na levels were used. Products missing Na data were excluded, and quality assurance steps were taken to remove implausible Na values. First, products with 0 mg of Na on the NFt but reporting salt or other Na-containing ingredients (e.g. monosodium glutamate) on the ingredients list were excluded. Second, outliers identified based on previously published Na ranges in the Canadian food supply^(12,15,17)^ were manually assessed for plausibility and excluded if considered implausible (e.g. error on the NFt or the retailer website for FLIP 2020).

### Statistical analysis

The distribution of Na (mg/100 g) including the 0th (minimum), 25th, 50th (median), 75th and 100th (maximum) percentiles, as well as the mean, was calculated for each year by subcategory. Quantile regression models were used to compare Na content at the 25th, 50th and 75th percentiles across each year-pair (i.e. 2010–2013, 2010–2017, 2010–2020, 2013–2017, 2013–2020 and 2017–2020). Products sold in multiple years are present in multiple collections of the FLIP database, meaning each year is not independent. However, prior analysis matching products between the 2013 and 2017 collections by Universal Product Code has found that most products were different (∼60 %) between consecutive collections^(17)^. Then, Na levels (mg/100 g) for each product were compared against the respective 2016 average target and maximum level^(12)^. Changes in the proportion of products meeting the average targets and maximum levels between each year-pair were assessed using Firth’s bias-reduced logistic regression models. Cochran–Armitage tests for trend were used to assess trends in the proportion of products meeting the average targets and maximum levels over time. These statistical methods are analogous to those employed in a repeated cross-sectional assessment of changes in the nutrient content of packaged foods and beverages after full implementation of the Chilean Food Labelling and Advertising Law^(24)^. Notably, the average Na levels (mg/100 g) for each category were not compared with the average Na targets because the average targets are sales weighted, and this study did not have access to sales data, which was prohibitively expensive. Statistical modelling or testing was not done for categories with a small sample size in any year (i.e. < 10 products). All statistical analyses were performed using R version 4.4.1 (R Core Team, 2024), and the significance level was *P* < 0·05.

Due to the expanded sampling of the FLIP 2020 database to include products from four additional stores that were not sampled in 2010, 2013 and 2017, a sensitivity analysis was conducted to compare Na distribution, using Kolmogorov–Smirnov tests, and the proportion of products meeting the SRT between the full 2020 sample (all seven stores) and the subset of products from only the three stores previously sampled. This analysis assessed whether adding products from the additional stores affected the Na levels observed in the 2020 dataset and informed our decision to include products from the additional stores.

## Results

**Figure 1.**
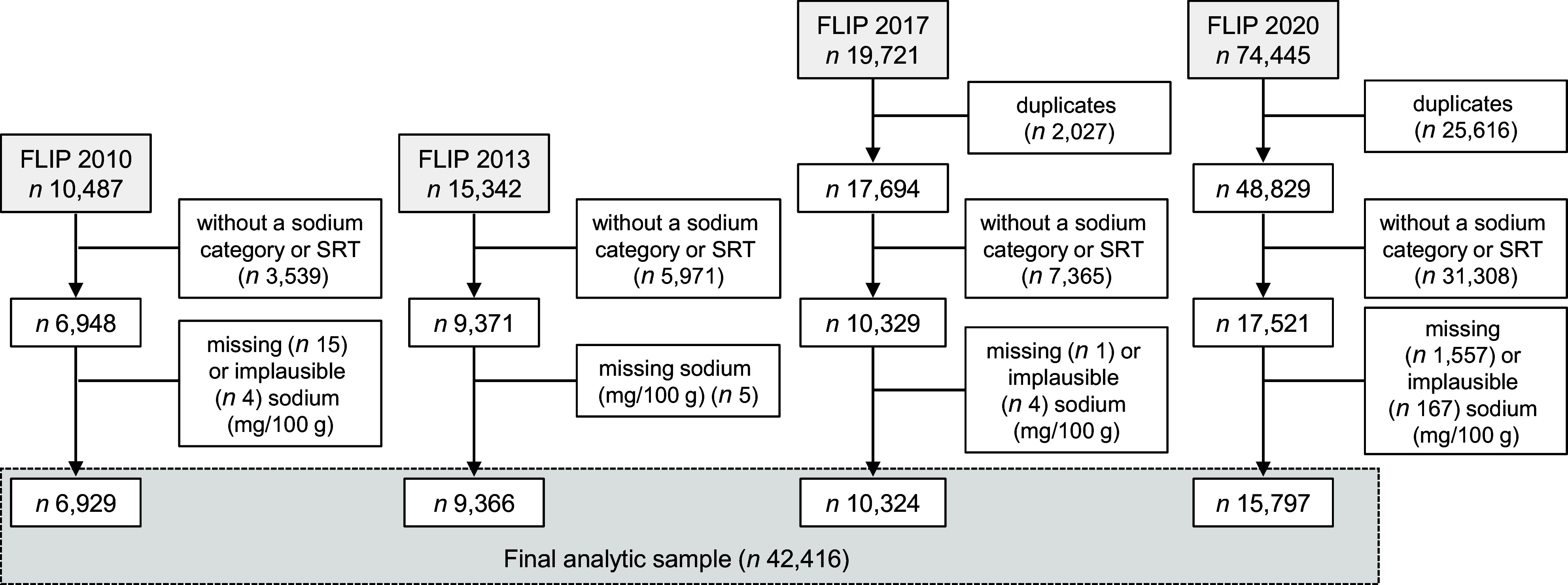
Exclusion flowcharts for the 2010, 2013, 2017 and 2020 collections of the FLIP database used to obtain the final sample of products for assessment of changes in sodium levels and comparison against Health Canada’s 2012–2016 voluntary SRT*. FLIP, Food Label Information and Price; SRT, sodium reduction targets. *Health Canada’s 2012–2016 voluntary SRT were published in the *Guidance for the Food Industry on Reducing Sodium in Processed Foods* document. Available at: https://www.canada.ca/en/health-canada/services/food-nutrition/legislation-guidelines/guidance-documents/guidance-food-industry-reducing-sodium-processed-foods-2012.html.

Product exclusions for each collection of the FLIP database are detailed in Figure 1. Products were excluded if they were duplicates (only collected in FLIP 2017 and 2020), did not fit into a Na category or have a SRT (i.e. low Na foods or foods that do not contribute substantially to Na intakes in Canada), were missing Na data or had implausible Na levels. The final analysis included 42 416 foods from 2010 (*n* 6929), 2013 (*n* 9366), 2017 (*n* 10 324) and 2020 (*n* 15 797) categorised into fourteen major, fifty-five sub and 192 minor categories. For FLIP 2020, products from all seven stores were included, as the sensitivity analysis showed no significant differences for any major category in Na distribution and only minor differences in the proportion of products meeting the SRT when the sample was restricted to only the three stores used in 2010, 2013 and 2017 (see online supplementary material, Supplemental Table 1).

### Changes in sodium distribution

Na distribution by year and major category are summarised in Table 1. Of the fourteen major categories there were thirteen with ≥ 10 products in all years. Between 2010 and 2013, 62 % (8/13) of major categories had a left shift (reduction) in any of the 25th, 50th or 75th percentiles of their Na distribution, 8 % (1/13) had a right shift (increase), 8 % (1/13) had both a right and left shift and 23 % (3/13) did not change. Between 2010 and 2017, 69 % (9/13) of major categories had a left shift, 15 % (2/13) had a right shift, 8 % (1/13) had both a left and right shift and 8 % (1/13) did not change. Overall, between 2010 and 2020, 54 % (7/13) of major categories had a left shift, 15 % (2/13) had a right shift, 15 % (2/13) had both a right and left shift and 15 % (2/13) did not change in their Na distribution. Between 2013 and 2017, 46 % (6/13) of major categories had a left shift, 23 % (3/13) had a right shift and 31 % (4/13) did not change. Between 2013 and 2020, 31 % (4/13) of major categories had a left shift, 31 % (4/13) had a right shift, 8 % (1/13) had both a right and left shift and 31 % (4/13) did not change. Between 2017 and 2020, 8 % (1/13) of major categories had a left shift, 46 % (6/13) had a right shift and 46 % (6/13) did not change. Detailed results by subcategory are reported in online supplementary material, Supplemental Table 2.

**Table 1.** Changes in the sodium content of Canadian processed foods between 2010, 2013, 2017 and 2020 by major category

			Mean sodium (mg/100 g)	Sodium percentiles (mg/100 g)				
Major category	Year	*n*		Min	25th	50th	75th	Max
Bakery products	2010	1768	449	0	265^bc^	400^abc^	548^bc^	2105
	2013	2222	433	0	257^e^	377^ade^	533^de^	1857
	2017	2935	400	0	247^bf^	350^bd^	500^bd^	3378
	2020	4097	400	0	235^cef^	348^ce^	508^ce^	2100
Breakfast cereals	2010	281	390	0	216^abc^	400^b^	556^abc^	933
	2013	309	319	0	100^ad^	350^d^	481^ad^	929
	2017	339	263	0	48^bdf^	230^bdf^	433^bdf^	867
	2020	755	327	0	118^cf^	370^f^	500^cf^	867
Dairy products and substitutes	2010	403	762	125	533	667^bc^	867^c^	2533
	2013	505	792	12	533	667^de^	867^e^	3000
	2017	612	762	20	567	700^bd^	800	2933
	2020	1059	719	27	553	700^ce^	779^ce^	3000
Fats and oils	2010	329	872	233	656^bc^	867^abc^	1000^bc^	1813
	2013	360	814	12	644	800^ade^	1000^de^	2600
	2017	410	727	19	600^b^	721^bd^	839^bd^	1728
	2020	682	740	17	600^c^	741^ce^	858^ce^	1818
Fish and seafood products	2010	287	444	53	300	413^bc^	540^c^	1436
	2013	397	540	27	291	388	536	8796
	2017	378	504	32	280	377^b^	500	8796
	2020	481	435	20	283	376^c^	500^c^	7600
Mixed dishes	2010	1202	357	0	258^abc^	325	430	1214
	2013	1515	353	6	244^a^	321	438^e^	1214
	2017	1237	349	3	248^b^	324	431	1067
	2020	1909	340	12	247^c^	316	417^e^	1182
Meat and meat substitutes	2010	594	835	49	553^abc^	800^abc^	1000^b^	3000
	2013	914	792	49	450^a^	705^ae^	960^d^	5000
	2017	962	760	53	427^bf^	667^bf^	879^bdf^	5020
	2020	1377	840	53	472^cf^	754^cef^	970^f^	3143
Soups	2010	333	270	33	224^ab^	260^a^	318	628
	2013	463	250	5	191^ade^	248^ae^	300^e^	536
	2017	491	253	2	203^bd^	249^f^	304^f^	632
	2020	865	261	0	211^e^	260^ef^	316^ef^	943
Snacks	2010	454	580	0	300	571^abc^	779^abc^	2080
	2013	722	522	0	270	500^a^	720^a^	2156
	2017	829	505	0	267	480^b^	697^b^	2020
	2020	1257	517	0	280	500^c^	700^c^	1933
Sauces, dips, gravies and condiments	2010	683	833	0	393^ab^	575^ab^	967	5800
	2013	1068	910	0	367^a^	527^a^	938^e^	9600
	2017	1154	931	0	360^bf^	504^bf^	1009	9333
	2020	1640	1032	0	383^f^	550^f^	1055^e^	10 214
Canned and bottled vegetables and legumes	2010	528	499	0	222^abc^	311^b^	588^abc^	2800
	2013	665	541	0	178^ade^	293^d^	800^a^	3500
	2017	617	530	0	99^bdf^	244^bdf^	800^b^	4667
	2020	972	605	0	146^cef^	328^f^	900^c^	4333
Seasoning mixes	2010	37	4529	33	1286	5250	6452^bc^	10 741
	2013	191	5913	0	2462	5333	7829^de^	30 000
	2017	203	7148	0	3042	6286	9512^bd^	30 000
	2020	392	7385	0	2667	6000	10 292^ce^	30 000
Infant and toddler foods^*^	2017	106	92	0	4	28^f^	143	500
	2020	233	104	0	19	78^f^	125	500
Salted nut butters	2010	30	273	0	102^ac^	333	400	467
	2013	35	343	109	300^ad^	357	433	667
	2017	51	300	109	222^df^	300^f^	371	533
	2020	78	344	67	303^cf^	367^f^	400	500

*Note:* Pairwise comparisons between the 25th, 50th and 75th percentile Na content for each year-pair (i.e. 2010–2013, 2010–2017, 2010–2020, 2013–2017, 2013–2020 and 2017–2020) were made using quantile regression models. A detailed analysis at the subcategory level is provided in online supplementary material, Supplemental Table 2.

* Infant and Toddler Foods were not collected in the FLIP 2010 and 2013 collections.

^a^ *P* < 0·05 for comparison between 2010 and 2013 from quantile regression.

^b^ *P* < 0·05 for comparison between 2010 and 2017 from quantile regression.

^c^ *P* < 0·05 for comparison between 2010 and 2020 from quantile regression.

^d^ *P* < 0·05 for comparison between 2013 and 2017 from quantile regression.

^e^ *P* < 0·05 for comparison between 2013 and 2020 from quantile regression.

^f^ *P* < 0·05 for comparison between 2017 and 2020 from quantile regression.

Left shifts occurred with similar frequencies across percentiles, with nine major categories showing a left shift at the 25th percentile, 9 at the 50th and 8 at the 75th, across all year comparisons. Right shifts also occurred with similar frequencies across percentiles, with 6 major categories showing a right shift at the 25th percentile, 8 at the 50th and 6 at the 75th.

### Changes in the proportion of products meeting Health Canada’s average targets and maximum levels

**Figure 2.**
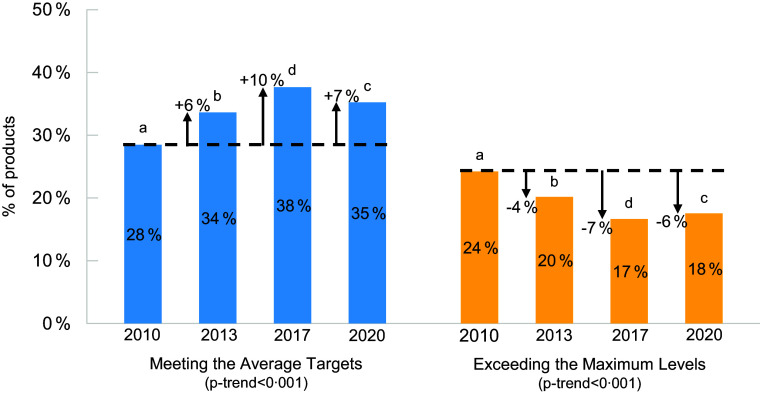
Proportion of processed foods meeting the average sodium reduction targets and exceeding the maximum sodium levels set by Health Canada for 2012–2016* in 2010, 2013, 2017 and 2020. Pairwise comparisons between proportions in each year-pair (i.e. 2010–2013, 2010–2017, 2010–2020, 2013–2017, 2013–2020 and 2017–2020) were made by contrasting estimated marginal means from Firth’s bias-reduced logistic regression model. The proportions were significantly different for all year-pair comparisons. *P*-trend was calculated using the Cochran–Armitage test. Data stratified by major category are presented in Table 2 and by subcategory in online supplementary material, Supplemental Table 1. *Health Canada’s 2012–2016 voluntary sodium reduction targets were published in the *Guidance for the Food Industry on Reducing Sodium in Processed Foods* document. Available at: https://www.canada.ca/en/health-canada/services/food-nutrition/legislation-guidelines/guidance-documents/guidance-food-industry-reducing-sodium-processed-foods-2012.html. ^a^ *P* < 0·05 for comparison between 2010 and all other years from logistic regression. ^b^ *P* < 0·05 for comparison between 2013 and all other years from logistic regression. ^c^ *P* < 0·05 for comparison between 2020 and all other years from logistic regression. ^d^ *P* < 0·05 for comparison between 2017 and all other years from logistic regression.

Changes in the proportion of products meeting the average targets and maximum levels are presented overall in Figure 2, by major category in Table 2 and by subcategory in online supplementary material, Supplemental Table 3. Overall, the proportion meeting the average targets increased slightly between 2010 (28 %) and 2013 (34 %; +6 %, *P* < 0·001) and between 2013 and 2017 (38 %; +4 %, *P* < 0·001), then decreased slightly between 2017 and 2020 (35 %; –3 %, *P* < 0·001), with a significant trend for increasing proportions (*P*-trend < 0·001) over time. Similarly, the proportion of products exceeding the maximum levels decreased slightly between 2010 (24 %) and 2013 (20 %; –4 %, *P* < 0·001) and between 2013 and 2017 (17 %, –3 %, *P* < 0·001), then increased between 2017 and 2020 (18 %; +1 %, *P* = 0·02) with a significant decreasing trend (*P*-trend < 0·001) over time.

**Table 2.** Proportion of processed foods meeting the average sodium reduction targets and exceeding the maximum sodium levels set by Health Canada for 2012–2016^*^ in 2010, 2013, 2017 and 2020 by major category

			Average target		Maximum level	
	Year	*n*	% Meeting average target	*P*-trend	% Exceeding maximum level	*P*-trend
Bakery products	2010	1768	30^bc^	< 0·001	23^abc^	< 0·001
	2013	2222	31^e^		18^a^	
	2017	2935	33^b^		16^b^	
	2020	4097	34^ce^		17^c^	
Breakfast cereals	2010	281	42^ab^	0·210	15^abc^	< 0·001
	2013	309	51^ad^		9^ad^	
	2017	339	63^bdf^		5^bd^	
	2020	755	48^f^		6^c^	
Dairy products and substitutes	2010	403	50	0·085	15	0·102
	2013	505	49		13	
	2017	612	47		12	
	2020	1059	46		11	
Fats and oils	2010	329	14^b^	0·129	22^b^	0·004
	2013	360	14^d^		25^de^	
	2017	410	20^bd^		15^bd^	
	2020	682	16		17^e^	
Fish and seafood products	2010	287	29	0·497	20^b^	0·054
	2013	397	30		19^d^	
	2017	378	31		12^bd^	
	2020	481	31		17	
Mixed dishes	2010	1202	28^a^	0·494	18^bc^	0·007
	2013	1515	34^a^		17	
	2017	1237	31		15^b^	
	2020	1909	31		15^c^	
Meat and meat substitutes	2010	594	30^abc^	< 0·001	61^abc^	< 0·001
	2013	914	40^ad^		46^ade^	
	2017	962	48^bdf^		39^bd^	
	2020	1377	43^cf^		41^ce^	
Soups	2010	333	29^abc^	0·486	12	0·549
	2013	463	44^ae^		11	
	2017	491	44^bf^		8	
	2020	865	36^cef^		11	
Snacks	2010	454	22^abc^	0·100	22^bc^	< 0·001
	2013	722	31^a^		18^e^	
	2017	829	31^b^		16^b^	
	2020	1257	28^c^		13^ce^	
Sauces, dips, gravies and condiments	2010	683	25^abc^	< 0·001	23^b^	0·542
	2013	1068	31^ad^		19^d^	
	2017	1154	37^bdf^		16^bdf^	
	2020	1640	32^cf^		21^f^	
Canned and bottled vegetables and legumes	2010	528	16^abc^	< 0·001	29^abc^	< 0·001
	2013	665	25^ade^		20^ade^	
	2017	617	39^bdf^		14^bd^	
	2020	972	32^cef^		15^ce^	
Seasoning mixes	2010	37	41	0·289	8	0·019
	2013	191	39		17	
	2017	203	48^f^		17	
	2020	392	35^f^		23	
Infant and toddler foods^†^	2017	106	81	0·373	6	0·339
	2020	233	85		3	
Salted nut butters	2010	30	43	0·262	17	0·518
	2013	35	29^d^		29^d^	
	2017	51	57^df^		10^d^	
	2020	78	26^f^		18	

*Note:* Pairwise comparisons between proportions for each year-pair (i.e. 2010–2013, 2010–2017, 2010–2020, 2013–2017, 2013–2020 and 2017–2020) were made by contrasting estimated marginal means from Firth’s bias-reduced logistic regression model. *P*-trend was calculated using Cochran–Armitage tests with significance level *P* < 0·05. Detailed analysis by subcategory is in online supplementary material, Supplemental Table 3.

* Health Canada’s 2012–2016 voluntary sodium reduction targets were published in the *Guidance for the Food Industry on Reducing Sodium in Processed Foods* document. Available at: https://www.canada.ca/en/health-canada/services/food-nutrition/legislation-guidelines/guidance-documents/guidance-food-industry-reducing-sodium-processed-foods-2012.html.

† Infant and Toddler Foods were not collected in the FLIP 2010 and 2013 collections.

^a^ *P* < 0·05 for comparison between 2010 and 2013 from logistic regression.

^b^ *P* < 0·05 for comparison between 2010 and 2017 from logistic regression.

^c^ *P* < 0·05 for comparison between 2010 and 2020 from logistic regression.

^d^ *P* < 0·05 for comparison between 2013 and 2017 from logistic regression.

^e^ *P* < 0·05 for comparison between 2013 and 2020 from logistic regression.

^f^ *P* < 0·05 for comparison between 2017 and 2020 from logistic regression.

Between 2010 and 2020, the proportion of products meeting the average targets increased in 46 % (6/13) of major categories and did not change in 54 % (7/13) of major categories using data for all 4 years. The largest increases in the proportion of products meeting the average targets were in canned and bottled vegetables and legumes (+16 %; 16 % in 2010 to 32 % in 2020) and meat and meat substitutes (+13 %; 30 % in 2010 to 43 % in 2020). Most increases in the proportion of products meeting the average targets occurred between 2010 and 2013 (7/13 major categories) and 2013 and 2017 (6/13). No major categories significantly increased the proportion of products meeting the average target between 2017 and 2020, rather the proportion decreased in 7/13 major categories. Most major categories (10/13) had the highest proportion of products meeting the average targets in 2017. Trend analysis showed an increasing trend in the proportion of products meeting the average targets for only 4/13 major categories.

Between 2010 and 2020, the proportion of foods exceeding the maximum levels decreased in 46 % (6/13) of major categories and did not change in 54 % (7/13). The largest reductions in the proportion of products exceeding the maximum levels were in meat and meat substitutes (–20 %; 61 % in 2010 to 41 % in 2020) and canned and bottled vegetables and legumes (–14 %; 29 % in 2010 to 15 % in 2020). Decreases in the proportion of products exceeding the maximum levels occurred between 2010 and 2013 (4/13 major categories) and 2013 and 2017 (7/13). No major categories decreased in the proportion of products exceeding the maximum levels between 2017 and 2020, rather, the proportion increased in 1/13 major categories. Most major categories (10/13) had the lowest proportion of products exceeding the maximum levels in 2017. Trend analysis showed a decreasing trend in the proportion of products exceeding the maximum levels for 7/13 major categories and an increasing trend for 1/13.

## Discussion

This study provides a comprehensive analysis of changes in the Na content of processed foods sold in Canada and changes in the proportion of products meeting Health Canada’s voluntary SRT between 2010 and 2020. It builds upon previous research showing limited progress^(15–18)^ and provides insights regarding the long-term impacts of Canada’s Na reduction initiatives which can help shape future initiatives or stronger measures. This analysis found limited progress in reducing Na levels between 2010 and 2020, finding just over half of major categories (54 %) shifted left in their Na distribution, fewer than half (46 %) increased the proportion of products meeting the average target and fewer than half (46 %) increased the proportion meeting the maximum levels. Additionally, between 2010 and 2020, many major categories (46 %) had no changes, a right shift or both a left and right shift in their Na distribution. Most Na reduction occurred between 2010 and 2017 with comparisons between 2017 and 2020 finding only one major category reduced in Na and many (46 %) increased. Similarly, the proportion of products meeting the average targets and maximum levels improved modestly between 2010 and 2017 then declined between 2017 and 2020. Overall, these findings indicate that limited progress was made to reduce Na in Canadian processed foods between 2010 and 2017, and no further progress occurred between 2017 and 2020, with evidence for some regression.

Although progress was limited, reductions in Na were observed in some important food categories. Bakery products and mixed dishes are the top two contributors to Canadians’ Na intakes^(8)^ and both had left shifts in their Na distribution between 2010 and 2020. In bakery products, Na decreased across the entire distribution, with left shifts at the 25th, 50th and 75th percentiles. However, both the magnitude of the Na reductions and the changes in the proportion of products meeting the SRT were modest over the 10 years. Simple average mean Na levels decreased 11 % for bakery products and 5 % for mixed dishes, although not directly comparable to the targeted sales-weighed reduction of 25–30 % for most categories. The proportion of products exceeding the maximum levels, thresholds for which no products should exceed, decreased 6 % in bakery products and 3 % in mixed dishes, with 17 % and 15 % of products still exceeding the maximums in 2020. The proportion of products meeting the average target increased 4 % in bakery products and 3 % in mixed dishes. These limited improvements indicate that, although the Na distributions of bakery products and mixed dishes shifted left, the reductions were not large enough to bring many products below the SRT and would be unlikely to have a significant impact on Na intakes. As the most recent assessment of Canadian’s Na intakes was from 2015^(8)^, an updated intake assessment based on current food composition data is needed to understand how changes in Na levels of processed foods translate into changes in Na intakes.

The finding that progress has been limited is consistent with assessments of voluntary Na reduction initiatives from other countries. Australia published voluntary SRT as maximum levels for twenty-seven food categories in 2020, with the aim of industry compliance by 2024. A two-year progress assessment found minimal Na reductions, with only 14 % of products lowering Na content by an average of 3 %^(25)^. Overall, the proportion of products meeting the SRT increased 6 % between 2020 and 2022, and the decrease in population Na intakes was estimated to be minimal at 0·3 % (8·3 mg/person/day)^(25)^. The Canadian data in this study also showed a 6 % increase in the proportion meeting the maximum levels, albeit over a 10-year period. The United States Food and Drug Administration published voluntary SRT for forty-seven packaged food categories in October 2021, with the goal of reducing Na consumption from 3400 mg to 3000 mg/d by April 2024^(26)^. The Phase 1 voluntary SRT were established using baseline data from 2010^(27)^; however, their publishing was delayed^(28)^. Analyses of progress between 2010 and 2022 found the sales-weighted average Na content in 60 % of packaged food categories decreased, 25 % increased and 13 % did not change^(27)^, resulting in a small, 3 % decrease in median sales-weighted Na content among packaged foods^(28)^. The Canadian data in this study, although not sales-weighted, found comparable changes between 2010 and 2020; a left shift in the Na distribution of most major categories (54 %) and a right shift or no shift in some major categories (15 % and 15 %, respectively) between 2010 and 2020.

Insights from the United Kingdom’s salt reduction program suggest that voluntary SRT can be effective at reducing Na levels in packaged foods when implemented alongside (1) complementary accountability initiatives that promote industry adherence to voluntary SRT and (2) frequent and transparent monitoring. The United Kingdom’s salt reduction program included setting progressively lower voluntary SRT every 2–3 years, political support with threats of regulation, a public health awareness campaign, frequent monitoring of Na levels in foods, praising companies that made progress, ‘naming and shaming’ companies not making progress and assessing Na intakes through 24 h urinary Na measurements every 3–5 years^(29)^. An evaluation of the program found a 15 % reduction in Na intakes and that many processed food categories had lower levels of Na^(29)^. For example, between 2001 and 2011, the salt content in pre-sliced bread decreased by 20 %, from 492 ± 76 mg/100 g to approximately 392 ± 52 mg/100 g^(29,30)^. In Canada, although starting later, over the 10 years between 2010 and 2020, there was no reduction in Na in pantry bread, one of the largest contributors to Na intakes, and mean Na levels actually increased by 4 % from 432 ± 97 mg/100 g in 2010 to 449 ± 124 mg/100 g in 2020 (data not shown for minor Na categories). In the United Kingdom, after reinvigoration of the salt reduction program in 2017, updated monitoring of Na levels in sliced breads found Na was further reduced to 360 mg/100 g in 2023, a decrease of 8 % from 2011^(31)^. Adding accountability initiatives like those implemented in the United Kingdom (e.g. political support with threats of legislation, a public health awareness campaign) and frequent and transparent monitoring (e.g. yearly assessment of Na levels in foods, assessment of Na intakes every few years, praising and shaming companies based on progress) to Canada’s current voluntary SRT can create a more comprehensive Na reduction program that provides a stronger incentive for manufacturers to reduce Na in processed foods.

This study found that while initial progress to reduce Na was made between 2010 and 2017, progress stopped between 2017 and 2020, coinciding with the gap between the 2012–2016 SRT and the updated 2020–2025 SRT. A similar pattern occurred in the United Kingdom, where Na reduction progress stalled after 2010^(32–34)^ due to government changes that disrupted the salt reduction program, placed responsibility for improving nutrition on the food industry and relaxed independent reporting mechanisms, relying instead on the food industry to self-report progress^(34)^. Together, this suggests that although industry may initially respond to voluntary initiatives, progress diminishes or stops without ongoing, independent efforts to encourage Na reduction, such as updated SRT, frequent and transparent monitoring and sustained pressure from the public, media and government.

Mandatory SRT, which more effectively reduce Na in packaged foods and reduce Na intakes, are recommended by the WHO and increasingly favoured over voluntary initiatives for their broader coverage and ability to create a level playing field^(1,35)^. South Africa set mandatory maximum Na levels for thirteen categories, and evaluations found the targets were effective, showing significant decreases in Na intakes^(36)^ and high compliance rates, with 70–75 % of products meeting the targets^(37)^. In Argentina, mandatory maximum Na levels for three food groups came into force in 2014, and subsequent evaluations found high compliance, with 94 % of products below the maximum in both 2017/2018 and again in 2022^(38,39)^. However, the Na contents of products included in the law changed minimally, likely because most products (∼85 %) already met the maximum levels before the law^(38,40)^ and modelling suggested that even 100 % compliance would not have a significant impact on reducing Na intakes^(41)^. These findings have resulted in calls for stricter targets^(38–40)^ and highlight the importance of setting mandatory SRT that are both feasible and result in meaningful reductions. Our findings suggest that if Canada were to mandate the maximum Na levels from the 2012–2016 SRT, most products (82 % in 2020) would already meet the maximum levels and modelling has shown that even full compliance with the stricter average targets would not achieve Na intake recommendations^(42)^. Experience from Argentina and South Africa has shown that some foods continue to exceed the maximum levels, suggesting that mandatory SRT still need to be accompanied by frequent monitoring and enforcement^(39)^. Overall, a mandatory approach to Na reduction (e.g. mandatory SRT) is more effective than voluntary approaches; however, it must be stringent enough to impact a large proportion of frequently consumed processed foods and, once implemented, requires monitoring and enforcement.

All WHO Member States, including Canada, committed to a goal of reducing population Na intake by 30 % by 2025^(1)^. In 2023, the WHO Global Report on Sodium Intake Reduction was published to assess Member States’ progress in implementing Na reduction measures and highlight areas where further action can be taken^(1)^. The report scored all Member States from 1 to 4 with Canada scoring 2/4. Due to the absence of mandatory measures, Canada scored lower than fifty two other Member States, including both high (e.g. United States, the United Kingdom and Chile) and middle income (e.g. Argentina, Islamic Republic of Iran and Malaysia) countries, suggesting that Canada’s Na reduction measures are falling behind.

In 2026, front-of-package (FOP) labelling will be mandatory in Canada, and foods high in Na, sugar and saturated fat will be required to display a ‘High in’ nutrition symbol^(43)^. FOP labelling will elevate Canada’s score from 2 to 3 on the WHO scoring system. FOP labelling regulations, which are primarily used to help consumers make informed food choices^(43,44)^, may also promote Na reformulation^(24,45)^. In Canada, the FOP labelling regulations may encourage Na reduction beyond what has been observed under the voluntary SRT. 54 % of products in FLIP 2020 that exceeded the 2012–2016 average Na targets would be required to carry a ‘high in’ symbol for Na under Health Canada’s FOP labelling regulations (see online supplementary material, Supplemental Figure 1)^(46,47)^. These products may be reformulated to avoid displaying an FOP symbol. The remaining 46 % of products that exceed the average target would not be required to display an FOP symbol for sodium and are unlikely to be reduced in Na in response to FOP labelling. To incentivise Na reduction in these products, the implementation of additional Na reduction measures—such as mandatory SRT or strong accountability initiatives to encourage alignment with voluntary SRT—would be important complementary policies to FOP labelling.

### Limitations

There are limitations of this study that should be considered. The average targets were established by Health Canada as sales-weighted averages. Sales weightings are used to better reflect consumption as frequently purchased foods have a larger influence on a sales-weighted average target than foods purchased less frequently. Due to prohibitive costs of purchasing sales data, the Na levels presented in this analysis are unweighted and as such, this study did not compare the simple averages to the sales-weighted average targets, instead focusing on changes in Na distribution and the proportion of products meeting the SRT. However, prior research comparing simple to sales-weighted average Na content has suggested that simple averages are relevant for most food categories^(17,48)^. Sales weighting also may inhibit transparent monitoring, as it requires sales data for all products in a manufacturer’s portfolio. Previous sales-weighted assessments have reported sales-weighted composites for foods in a category^(16,18)^ rather than per manufacturer, as is the aim for the average targets for manufacturers^(12,13)^. This approach obscures progress of individual products and manufacturers. Therefore, the benefit of sales-weightings should be considered against its added costs and the challenge it poses for transparent monitoring.

Another consideration is differences in data collection methodologies and scope between the FLIP 2010, 2013 and 2017 collections and the 2020 collection. All label data in 2010, 2013 and 2017 were collected in person at three or four top grocery retailers (i.e. Loblaws, Metro, Sobeys and Safeway; Safeway was only collected in 2010 and 2013 as the company was acquired by Sobeys)^(19–21)^. Due to the COVID-19 pandemic, in-person data collection was not possible in 2020, and web scraping was used to collect label information from major grocery retailer websites. Web scraping also enabled data collection to expand to include seven grocery retailers (i.e. Loblaws, Metro, Voilà by Sobeys, Grocery Gateway by Longo’s, Costco, No Frills and Walmart)^(22)^. It is possible that differences in data collection impacted comparisons between years. In this study, we justified the inclusion of products from all seven retailers through a sensitivity analysis, which found no differences in Na distribution for any major category and only minor differences in the proportion of products meeting the SRT when the sample was restricted to the three stores sampled in 2010, 2013 and 2017 (see online supplementary material, Supplemental Table 1). Additionally, all four collections relied on NFt data for Na levels and serving sizes. Nutrient declarations on food packages are used globally for similar analyses, and one Canadian study found that a relatively small proportion of products had inaccurate Na content on the NFt^(49)^.

Limitations due to the study design should also be acknowledged. The repeated, cross-sectional design does not enable the establishment of causation, and we cannot conclude that observed changes in Na levels are due to the voluntary SRT. There are other factors that influence Na levels in products, such as changes in consumer behaviour, the supply chain or global policies, which were not controlled for. Additionally, it was not possible to assess how much of the observed changes in Na were attributable to reformulation *v*. product turnover (i.e. the addition and removal of foods between collections). However, previous analyses of matched products across collections in the FLIP database have shown that Na reformulation occurred infrequently and resulted in little or no significant reductions in Na, suggesting that observed reductions are more often due to product turnover^(17,50)^. Finally, this analysis did not assess changes in Canadians’ Na intakes. While processed foods are the primary source of Na in Canadians’ diets, behavioural changes also affect intake, and monitoring intakes is essential for tracking progress towards Canada’s commitment to a 30 % reduction in Na intakes.

### Conclusions

Overall, this study found that just over half of food categories decreased in Na content between 2010 and 2020; however, many did not change or increased. Minor improvements in the proportion of products meetings Health Canada’s goal average targets and maximum levels occurred between 2010 and 2017, suggesting the reductions in Na levels are unlikely to be nutritionally significant, and no progress was made between 2017 and 2020. Our findings reinforce results from prior assessments, which have identified that progress towards Na reduction has been minimal in Canada under voluntary initiatives. Further actions, such as implementing accountability initiatives that promote industry adherence to voluntary SRT or introducing mandatory Na reduction measures, alongside frequent, transparent monitoring are needed in Canada for manufacturers to reduce Na in processed foods.

## Supporting information

Ziraldo et al. supplementary material Ziraldo et al. supplementary material

## Data Availability

The corresponding author can be contacted with any questions regarding the data used in analysis; data may be shared subject to relevant policies of the host organisation.

## Supplementary material

For supplementary material accompanying this paper visit https://doi.org/10.1017/S1368980026102067
