# Supplementary material for: Sodium in Canadian processed foods between 2010 and 2020: implications for future sodium reduction initiatives
Source: Public Health Nutr. 2026 Feb 20;29(1):e52. doi: 10.1017/S1368980026102067 (PMC13112298; doi:10.1017/S1368980026102067)
Supplement: Ziraldo et al. supplementary material [file S1368980026102067sup001.docx]

**Supplementary Table 1.** Sensitivity analysis comparing sodium levels and adherence to Health Canada’s sodium reduction targets* in products collected in 2020 from all seven grocery stores and in products collected in 2020 from only the three stores previously sampled in 2010, 2013, and 2017.

|  | **n** | | **Median sodium (mg/100 g)** | | |  | | **% Meeting Average Target** | | | **% Exceeding Maximum Level** | | |
| --- | --- | --- | --- | --- | --- | --- | --- | --- | --- | --- | --- | --- | --- |
| **Category** | **Seven stores** | **Three stores** | **Seven stores** | **Three stores** | **Absolute % difference** | **p-value** | **Seven stores** | | **Three stores** | **Absolute difference** | **Seven stores** | **Three stores** | **Absolute difference** |
| **Bakery Products** | **4097** | **1983** | **348** | **348** | **0.0** | **0.642** | **34%** | | **32%** | **2%** | **17%** | **18%** | **1%** |
|  |  |  |  |  |  |  |  | |  |  |  |  |  |
| Bread products | 674 | 294 | 431 | 432 | 0.2 | 0.956 | 20% | | 18% | 2% | 24% | 23% | 1% |
|  |  |  |  |  |  |  |  | |  |  |  |  |  |
| Cookies | 844 | 375 | 280 | 286 | 2.1 | 0.506 | 38% | | 34% | 4% | 15% | 16% | 1% |
|  |  |  |  |  |  |  |  | |  |  |  |  |  |
| Toaster pastries | 39 | 22 | 320 | 333 | 4.1 | 0.992 | 54% | | 45% | 9% | 23% | 27% | 4% |
|  |  |  |  |  |  |  |  | |  |  |  |  |  |
| Tortillas and wraps | 86 | 39 | 556 | 557 | 0.2 | 0.971 | 49% | | 46% | 3% | 9% | 8% | 1% |
|  |  |  |  |  |  |  |  | |  |  |  |  |  |
| Granola bars | 792 | 448 | 237 | 240 | 1.3 | 1.000 | 34% | | 34% | 0% | 18% | 19% | 1% |
|  |  |  |  |  |  |  |  | |  |  |  |  |  |
| Dough and pastry | 73 | 27 | 438 | 500 | 14.2 | 0.886 | 27% | | 19% | 9% | 14% | 19% | 5% |
|  |  |  |  |  |  |  |  | |  |  |  |  |  |
| Crackers | 553 | 274 | 667 | 671 | 0.6 | 1.000 | 41% | | 41% | 0% | 18% | 18% | 0% |
|  |  |  |  |  |  |  |  | |  |  |  |  |  |
| Dry bread | 173 | 74 | 667 | 724 | 8.5 | 0.570 | 22% | | 15% | 7% | 21% | 23% | 2% |
|  |  |  |  |  |  |  |  | |  |  |  |  |  |
| Breadcrumbs, croutons and salad toppers | 95 | 48 | 714 | 767 | 7.4 | 0.994 | 47% | | 48% | 1% | 12% | 13% | 1% |
|  |  |  |  |  |  |  |  | |  |  |  |  |  |
| Commercial and frozen baked desserts | 615 | 289 | 284 | 304 | 7.0 | 0.151 | 34% | | 29% | 5% | 15% | 17% | 2% |
|  |  |  |  |  |  |  |  | |  |  |  |  |  |
| Breakfast foods (e.g., pancakes, waffles, scones) | 153 | 93 | 471 | 469 | 0.4 | 0.998 | 37% | | 35% | 2% | 5% | 4% | 1% |
|  |  |  |  |  |  |  |  | |  |  |  |  |  |
| **Breakfast Cereals** | **755** | **406** | **370** | **359** | **3.0** | **0.882** | **48%** | | **50%** | **2%** | **6%** | **6%** | **0%** |
|  |  |  |  |  |  |  |  | |  |  |  |  |  |
| Ready-to-eat-cereals | 651 | 353 | 375 | 370 | 1.3 | 0.988 | 46% | | 47% | 1% | 7% | 7% | 0% |
|  |  |  |  |  |  |  |  | |  |  |  |  |  |
| Hot instant cereals | 104 | 53 | 280 | 200 | 28.6 | 0.638 | 60% | | 66% | 6% | 0% | 0% | 0% |
|  |  |  |  |  |  |  |  | |  |  |  |  |  |
| **Dairy Products and Substitutes** | **1059** | **595** | **700** | **700** | **0.0** | **0.924** | **46%** | | **44%** | **2%** | **11%** | **12%** | **1%** |
|  |  |  |  |  |  |  |  | |  |  |  |  |  |
| Cheese | 945 | 530 | 667 | 680 | 1.9 | 0.919 | 47% | | 46% | 1% | 12% | 12% | 0% |
|  |  |  |  |  |  |  |  | |  |  |  |  |  |
| Processed cheese and other cheese products | 79 | 44 | 1333 | 1491 | 11.9 | 0.141 | 47% | | 30% | 17% | 8% | 14% | 6% |
|  |  |  |  |  |  |  |  | |  |  |  |  |  |
| Dairy-free cheese and spreads | 35 | 21 | 800 | 821 | 2.6 | 0.633 | 14% | | 19% | 5% | 3% | 5% | 2% |
|  |  |  |  |  |  |  |  | |  |  |  |  |  |
| **Fats and Oils** | **682** | **412** | **741** | **750** | **1.2** | **0.990** | **16%** | | **14%** | **2%** | **17%** | **17%** | **0%** |
|  |  |  |  |  |  |  |  | |  |  |  |  |  |
| Salted margarine | 82 | 54 | 650 | 650 | 0.0 | 0.969 | 6% | | 6% | 0% | 4% | 4% | 0% |
|  |  |  |  |  |  |  |  | |  |  |  |  |  |
| Salad dressing and mayonnaise | 525 | 315 | 774 | 784 | 1.3 | 0.805 | 20% | | 17% | 3% | 21% | 20% | 1% |
|  |  |  |  |  |  |  |  | |  |  |  |  |  |
| Salted butter and dairy spreads | 75 | 43 | 700 | 600 | 14.3 | 0.336 | 0% | | 0% | 0% | 5% | 7% | 2% |
|  |  |  |  |  |  |  |  | |  |  |  |  |  |
| **Fish and Seafood Products** | **481** | **193** | **376** | **368** | **2.1** | **0.972** | **31%** | | **30%** | **1%** | **17%** | **15%** | **2%** |
|  |  |  |  |  |  |  |  | |  |  |  |  |  |
| Canned tuna | 84 | 37 | 392 | 350 | 10.7 | 0.944 | 11% | | 16% | 5% | 33% | 30% | 3% |
|  |  |  |  |  |  |  |  | |  |  |  |  |  |
| Imitation seafood | 6 | 3 | 400 | 330 | 17.5 | n/a | 100% | | 100% | 0% | 0% | 0% | 0% |
|  |  |  |  |  |  |  |  | |  |  |  |  |  |
| Canned fish and seafood | 196 | 91 | 368 | 388 | 5.4 | 0.989 | 34% | | 30% | 4% | 16% | 19% | 3% |
|  |  |  |  |  |  |  |  | |  |  |  |  |  |
| Frozen fish and seafood | 191 | 61 | 376 | 345 | 8.2 | 0.795 | 36% | | 36% | 0% | 9% | 0% | 9% |
|  |  |  |  |  |  |  |  | |  |  |  |  |  |
| Seafood spread and dips | 4 | 1 | 4527 | 7600 | 67.9 | n/a | 0% | | 0% | 0% | 100% | 100% | 0% |
|  |  |  |  |  |  |  |  | |  |  |  |  |  |
| **Mixed Dishes** | **1909** | **947** | **316** | **307** | **2.8** | **0.319** | **31%** | | **32%** | **1%** | **15%** | **15%** | **0%** |
|  |  |  |  |  |  |  |  | |  |  |  |  |  |
| Canned and dry foods as consumed | 519 | 323 | 292 | 289 | 1.0 | 0.998 | 36% | | 34% | 2% | 13% | 13% | 0% |
|  |  |  |  |  |  |  |  | |  |  |  |  |  |
| Refrigerated and frozen appetizers, sides and entrees | 893 | 375 | 310 | 280 | 9.7 | 0.035^†^ | 32% | | 34% | 2% | 18% | 17% | 1% |
|  |  |  |  |  |  |  |  | |  |  |  |  |  |
| Pizza, pizza snacks and frozen sandwiches | 246 | 117 | 489 | 462 | 5.5 | 0.571 | 15% | | 20% | 5% | 6% | 3% | 3% |
|  |  |  |  |  |  |  |  | |  |  |  |  |  |
| Potatoes | 159 | 96 | 268 | 271 | 1.1 | 0.952 | 36% | | 32% | 4% | 23% | 26% | 3% |
|  |  |  |  |  |  |  |  | |  |  |  |  |  |
| Refrigerated prepared salads | 92 | 36 | 295 | 320 | 8.5 | 0.446 | 25% | | 25% | 0% | 13% | 11% | 2% |
|  |  |  |  |  |  |  |  | |  |  |  |  |  |
| **Meat and Meat Substitutes** | **1377** | **595** | **754** | **730** | **3.2** | **0.384** | **43%** | | **45%** | **2%** | **41%** | **43%** | **2%** |
|  |  |  |  |  |  |  |  | |  |  |  |  |  |
| Bacon and substitutes | 57 | 29 | 611 | 611 | 0.0 | 1.000 | 58% | | 66% | 8% | 30% | 21% | 9% |
|  |  |  |  |  |  |  |  | |  |  |  |  |  |
| Packaged sausages and wieners | 225 | 93 | 792 | 760 | 4.0 | 0.133 | 47% | | 54% | 7% | 31% | 31% | 0% |
|  |  |  |  |  |  |  |  | |  |  |  |  |  |
| Packaged deli meats | 375 | 153 | 937 | 891 | 4.9 | 0.279 | 33% | | 37% | 4% | 53% | 51% | 2% |
|  |  |  |  |  |  |  |  | |  |  |  |  |  |
| Canned meat and poultry | 56 | 19 | 833 | 842 | 1.1 | 0.827 | 38% | | 26% | 12% | 61% | 74% | 13% |
|  |  |  |  |  |  |  |  | |  |  |  |  |  |
| Meat sticks and jerky | 117 | 60 | 1586 | 1733 | 9.3 | 0.009^†^ | 42% | | 32% | 10% | 55% | 63% | 8% |
|  |  |  |  |  |  |  |  | |  |  |  |  |  |
| Meat and poultry products | 395 | 184 | 421 | 434 | 3.1 | 0.956 | 57% | | 55% | 2% | 35% | 40% | 5% |
|  |  |  |  |  |  |  |  | |  |  |  |  |  |
| Bacon bits and shelf stable pre-cooked bacon | 19 | 11 | 2143 | 2500 | 16.7 | 0.991 | 32% | | 27% | 5% | 5% | 0% | 5% |
|  |  |  |  |  |  |  |  | |  |  |  |  |  |
| Meat substitutes | 133 | 46 | 451 | 475 | 5.3 | 0.980 | 24% | | 30% | 6% | 32% | 33% | 1% |
|  |  |  |  |  |  |  |  | |  |  |  |  |  |
| **Soups** | **865** | **591** | **260** | **259** | **0.4** | **1.000** | **36%** | | **36%** | **0%** | **11%** | **10%** | **1%** |
|  |  |  |  |  |  |  |  | |  |  |  |  |  |
| Bouillon and broth products as consumed | 205 | 135 | 243 | 243 | 0.0 | 0.998 | 47% | | 49% | 2% | 22% | 19% | 3% |
|  |  |  |  |  |  |  |  | |  |  |  |  |  |
| Canned condensed wet soup as consumed | 125 | 91 | 266 | 258 | 3.0 | 1.000 | 34% | | 35% | 1% | 2% | 2% | 0% |
|  |  |  |  |  |  |  |  | |  |  |  |  |  |
| Ready-to-serve soup | 254 | 170 | 256 | 257 | 0.4 | 0.933 | 32% | | 29% | 3% | 2% | 2% | 0% |
|  |  |  |  |  |  |  |  | |  |  |  |  |  |
| Fresh and instant oriental noodle soups as consumed | 202 | 138 | 286 | 286 | 0.0 | 1.000 | 38% | | 41% | 3% | 15% | 16% | 1% |
|  |  |  |  |  |  |  |  | |  |  |  |  |  |
| Dry soup mixes as consumed | 79 | 57 | 273 | 273 | 0.0 | 0.999 | 16% | | 16% | 0% | 10% | 12% | 2% |
|  |  |  |  |  |  |  |  | |  |  |  |  |  |
| **Snacks** | **1257** | **676** | **500** | **500** | **0.0** | **0.998** | **28%** | | **26%** | **2%** | **13%** | **12%** | **1%** |
|  |  |  |  |  |  |  |  | |  |  |  |  |  |
| Snack foods | 914 | 495 | 600 | 600 | 0.0 | 0.991 | 23% | | 22% | 1% | 10% | 9% | 1% |
|  |  |  |  |  |  |  |  | |  |  |  |  |  |
| Salted snacking fruits, nuts and seeds | 222 | 98 | 300 | 290 | 3.3 | 0.992 | 55% | | 58% | 3% | 16% | 12% | 4% |
|  |  |  |  |  |  |  |  | |  |  |  |  |  |
| Pudding as consumed | 121 | 83 | 126 | 126 | 0.0 | 1.000 | 17% | | 16% | 1% | 30% | 28% | 2% |
|  |  |  |  |  |  |  |  | |  |  |  |  |  |
| **Sauces, Dips, Gravies, and Condiments** | **1640** | **835** | **550** | **535** | **2.7** | **0.348** | **32%** | | **32%** | **0%** | **21%** | **19%** | **2%** |
|  |  |  |  |  |  |  |  | |  |  |  |  |  |
| Tomato sauce | 50 | 30 | 247 | 246 | 0.4 | 0.997 | 52% | | 53% | 1% | 0% | 0% | 0% |
|  |  |  |  |  |  |  |  | |  |  |  |  |  |
| Other sauces, dips, gravies, and condiments | 1309 | 623 | 673 | 674 | 0.1 | 0.772 | 30% | | 28% | 2% | 24% | 23% | 1% |
|  |  |  |  |  |  |  |  | |  |  |  |  |  |
| Pasta sauce | 281 | 182 | 355 | 349 | 1.7 | 0.922 | 40% | | 43% | 3% | 9% | 8% | 1% |
|  |  |  |  |  |  |  |  | |  |  |  |  |  |
| **Canned and Bottled Vegetables and Legumes**‡ | **972** | **504** | **328** | **283** | **13.7** | **0.327** | **32%** | | **35%** | **3%** | **15%** | **13%** | **2%** |
|  |  |  |  |  |  |  |  | |  |  |  |  |  |
| **Seasoning Mixes** | **392** | **214** | **6000** | **5526** | **7.9** | **0.818** | **35%** | | **37%** | **2%** | **23%** | **20%** | **3%** |
|  |  |  |  |  |  |  |  | |  |  |  |  |  |
| Breading, batter and coating mixes | 43 | 27 | 2667 | 2620 | 1.8 | 0.998 | 12% | | 15% | 3% | 28% | 30% | 2% |
|  |  |  |  |  |  |  |  | |  |  |  |  |  |
| Seasoning | 349 | 187 | 7000 | 6500 | 7.1 | 0.807 | 38% | | 40% | 2% | 22% | 19% | 3% |
|  |  |  |  |  |  |  |  | |  |  |  |  |  |
| **Infant and Toddler Foods** | **233** | **148** | **78** | **74** | **5.1** | **0.553** | **85%** | | **84%** | **1%** | **3%** | **3%** | **0%** |
|  |  |  |  |  |  |  |  | |  |  |  |  |  |
| Cookies, biscuits and snack bars | 103 | 66 | 39 | 17 | 56.4 | 0.794 | 73% | | 71% | 2% | 8% | 8% | 0% |
|  |  |  |  |  |  |  |  | |  |  |  |  |  |
| Mixed dishes | 90 | 59 | 82 | 82 | 0.0 | 0.123 | 97% | | 97% | 0% | 0% | 0% | 0% |
|  |  |  |  |  |  |  |  | |  |  |  |  |  |
| Savoury snacks | 40 | 23 | 71 | 71 | 0.0 | 0.993 | 90% | | 91% | 1% | 0% | 0% | 0% |
|  |  |  |  |  |  |  |  | |  |  |  |  |  |
| **Salted Nut Butters‡** | **78** | **43** | **367** | **367** | **0.0** | **0.935** | **26%** | | **30%** | **4%** | **18%** | **23%** | **5%** |
| **OVERALL** | **15,797** | **8,142** | **--** | **--** | **--** | **--** | **35%** | | **35%** | **0%** | **18%** | **17%** | **1%** |

**Note**: Comparisons of the sodium distribution among products collected in 2020 from all seven grocery stores (i.e., Loblaws, Metro, Voilà by Sobeys, Grocery Gateway by Longo’s, Costco, No Frills and Walmart) to the sodium distribution of products collected in 2020 from only the three stores sampled in previous collections in 2010, 2013, and 2017 (i.e., Loblaws, Metro, Voilà by Sobeys) were made using Kolmogorov-Smirnov tests. n/a, Kolmogorov-Smirnov test not conducted for categories with <10 products.

^*^Health Canada’s 2012-2016 voluntary sodium reduction targets were published in the Guidance for the Food Industry on Reducing Sodium in Processed Foods document. Available at: <https://www.canada.ca/en/health-canada/services/food-nutrition/legislation-guidelines/guidance-documents/guidance-food-industry-reducing-sodium-processed-foods-2012.html>.

^†^Significance level p<0.05.

^‡^There is only one subcategory under the canned and bottled vegetables and legumes and salted nut butters major categories.

**Supplementary Table 2**. Changes in the sodium content of Canadian processed foods between 2010, 2013, 2017, and 2020 by major category and subcategory.

|  |  |  | **Mean sodium (mg/100 g)** | **Sodium percentiles (mg/100g)** | | | | |
| --- | --- | --- | --- | --- | --- | --- | --- | --- |
| **Category** | **Year** | **n** |  | **Min** | **25th** | **50th** | **75th** | **Max** |
| **Bakery Products** | **2010** | **1768** | **449** | **0** | **265^bc^** | **400^abc^** | **548^bc^** | **2105** |
|  | **2013** | **2222** | **433** | **0** | **257^e^** | **377^ade^** | **533^de^** | **1857** |
|  | **2017** | **2935** | **400** | **0** | **247^bf^** | **350^bd^** | **500^bd^** | **3378** |
|  | **2020** | **4097** | **400** | **0** | **235^cef^** | **348^ce^** | **508^ce^** | **2100** |
|  |  |  |  |  |  |  |  |  |
| Bread Products | 2010 | 379 | 449 | 11 | 371 | 446^a^ | 520 | 976 |
|  | 2013 | 444 | 417 | 0 | 350 | 413^ad^ | 507^de^ | 750 |
|  | 2017 | 656 | 446 | 11 | 357 | 435^d^ | 529^d^ | 1060 |
|  | 2020 | 674 | 450 | 11 | 366 | 431 | 533^e^ | 1333 |
|  |  |  |  |  |  |  |  |  |
| Cookies | 2010 | 309 | 299 | 18 | 189 | 297 | 400^abc^ | 900 |
|  | 2013 | 397 | 277 | 0 | 185 | 286 | 360^a^ | 667 |
|  | 2017 | 543 | 281 | 25 | 200 | 283 | 348^b^ | 706 |
|  | 2020 | 844 | 276 | 0 | 183 | 280 | 348^c^ | 1111 |
|  |  |  |  |  |  |  |  |  |
| Toaster pastries | 2010 | 12 | 364 | 320 | 320^c^ | 352 | 385 | 460 |
|  | 2013 | 11 | 344 | 315 | 315^e^ | 320 | 352 | 440 |
|  | 2017 | 11 | 355 | 315 | 327^f^ | 333 | 367 | 440 |
|  | 2020 | 39 | 342 | 250 | 286^cef^ | 320 | 400 | 600 |
|  |  |  |  |  |  |  |  |  |
| Tortillas and wraps | 2010 | 27 | 603 | 20 | 519 | 667^bc^ | 735^b^ | 827 |
|  | 2013 | 63 | 647 | 246 | 594 | 680^de^ | 743^de^ | 881 |
|  | 2017 | 52 | 541 | 94 | 508 | 529^bd^ | 583^bdf^ | 906 |
|  | 2020 | 86 | 569 | 0 | 517 | 556^ce^ | 665^ef^ | 1033 |
|  |  |  |  |  |  |  |  |  |
| Granola bars | 2010 | 172 | 279 | 13 | 200^bc^ | 269^abc^ | 333 | 553 |
|  | 2013 | 199 | 254 | 16 | 187^de^ | 243^a^ | 304 | 553 |
|  | 2017 | 379 | 232 | 0 | 132^bd^ | 233^b^ | 320 | 900 |
|  | 2020 | 792 | 238 | 0 | 140^ce^ | 237^c^ | 320 | 771 |
|  |  |  |  |  |  |  |  |  |
| Dough and pastry | 2010 | 29 | 507 | 246 | 318 | 429 | 679 | 1074 |
|  | 2013 | 47 | 499 | 172 | 314 | 431 | 673 | 1154 |
|  | 2017 | 59 | 495 | 172 | 319 | 435 | 593 | 1038 |
|  | 2020 | 73 | 463 | 6 | 281 | 438 | 548 | 1059 |
|  |  |  |  |  |  |  |  |  |
| Crackers | 2010 | 268 | 749 | 0 | 500 | 676 | 951^bc^ | 2105 |
|  | 2013 | 301 | 738 | 0 | 525 | 700 | 900 | 1857 |
|  | 2017 | 317 | 680 | 0 | 474 | 650 | 842^b^ | 2000 |
|  | 2020 | 553 | 698 | 0 | 500 | 667 | 850^c^ | 2050 |
|  |  |  |  |  |  |  |  |  |
| Dry bread | 2010 | 65 | 656 | 0 | 500 | 725 | 864 | 1399 |
|  | 2013 | 107 | 666 | 0 | 488 | 675 | 854 | 1250 |
|  | 2017 | 117 | 655 | 0 | 409 | 625 | 800 | 3378 |
|  | 2020 | 173 | 680 | 0 | 500 | 667 | 818 | 1700 |
|  |  |  |  |  |  |  |  |  |
| Breadcrumbs, croutons and salad toppers | 2010 | 56 | 886 | 133 | 714^bc^ | 929^c^ | 1143^c^ | 1357 |
|  | 2013 | 64 | 895 | 133 | 593 | 904 | 1163^e^ | 1800 |
|  | 2017 | 88 | 791 | 0 | 500^b^ | 786 | 1013 | 2100 |
|  | 2020 | 95 | 721 | 117 | 400^c^ | 714^c^ | 939^ce^ | 2100 |
|  |  |  |  |  |  |  |  |  |
| Commercial and frozen baked desserts | 2010 | 383 | 307 | 25 | 193 | 286 | 393^bc^ | 1408 |
|  | 2013 | 478 | 298 | 18 | 204 | 300^d^ | 371^d^ | 956 |
|  | 2017 | 613 | 273 | 15 | 207 | 273^d^ | 341^bd^ | 915 |
|  | 2020 | 615 | 284 | 20 | 205 | 284 | 354^c^ | 822 |
|  |  |  |  |  |  |  |  |  |
| Breakfast foods (e.g., pancakes, waffles, scones) | 2010 | 68 | 558 | 208 | 471^ab^ | 543^c^ | 634^c^ | 844 |
|  | 2013 | 111 | 509 | 196 | 420^a^ | 514^e^ | 608^e^ | 812 |
|  | 2017 | 100 | 497 | 180 | 394^b^ | 500 | 574^f^ | 1378 |
|  | 2020 | 153 | 463 | 214 | 429 | 471^ce^ | 529^cef^ | 700 |
|  |  |  |  |  |  |  |  |  |
| **Breakfast Cereals** | **2010** | **281** | **390** | **0** | **216^abc^** | **400^b^** | **556^abc^** | **933** |
|  | **2013** | **309** | **319** | **0** | **100^ad^** | **350^d^** | **481^ad^** | **929** |
|  | **2017** | **339** | **263** | **0** | **48^bdf^** | **230^bdf^** | **433^bdf^** | **867** |
|  | **2020** | **755** | **327** | **0** | **118^cf^** | **370^f^** | **500^cf^** | **867** |
|  |  |  |  |  |  |  |  |  |
| Ready-to-eat-cereals | 2010 | 230 | 375 | 0 | 173^ab^ | 383^ab^ | 547^ab^ | 933 |
|  | 2013 | 250 | 301 | 0 | 82^ae^ | 255^ae^ | 477^a^ | 929 |
|  | 2017 | 256 | 279 | 0 | 91^bf^ | 243^bf^ | 434^bf^ | 867 |
|  | 2020 | 651 | 340 | 0 | 141^ef^ | 375^ef^ | 523^f^ | 867 |
|  |  |  |  |  |  |  |  |  |
| Hot instant cereals | 2010 | 51 | 455 | 200 | 319^bc^ | 469^bc^ | 576^abc^ | 711 |
|  | 2013 | 59 | 392 | 0 | 300^de^ | 442^de^ | 475^ae^ | 778 |
|  | 2017 | 83 | 212 | 0 | 0^bd^ | 200^bd^ | 409^b^ | 571 |
|  | 2020 | 104 | 241 | 0 | 0^ce^ | 280^ce^ | 442^ce^ | 571 |
|  |  |  |  |  |  |  |  |  |
| **Dairy Products and Substitutes** | **2010** | **403** | **762** | **125** | **533** | **667^bc^** | **867^c^** | **2533** |
|  | **2013** | **505** | **792** | **12** | **533** | **667^de^** | **867^e^** | **3000** |
|  | **2017** | **612** | **762** | **20** | **567** | **700^bd^** | **800** | **2933** |
|  | **2020** | **1059** | **719** | **27** | **553** | **700^ce^** | **779^ce^** | **3000** |
|  |  |  |  |  |  |  |  |  |
| Cheese | 2010 | 348 | 659 | 125 | 500 | 667 | 733 | 2533 |
|  | 2013 | 437 | 694 | 12 | 500 | 667 | 733 | 3000 |
|  | 2017 | 540 | 696 | 20 | 532 | 667 | 762 | 2933 |
|  | 2020 | 945 | 678 | 27 | 526 | 667 | 750 | 3000 |
|  |  |  |  |  |  |  |  |  |
| Processed cheese and other cheese products | 2010 | 49 | 1471 | 310 | 1381^bc^ | 1581^c^ | 1667^c^ | 2000 |
|  | 2013 | 56 | 1525 | 788 | 1395^de^ | 1619^e^ | 1758^e^ | 1905 |
|  | 2017 | 60 | 1352 | 652 | 900^bd^ | 1488 | 1645^f^ | 1905 |
|  | 2020 | 79 | 1191 | 317 | 879^ce^ | 1333^ce^ | 1526^cef^ | 1905 |
|  |  |  |  |  |  |  |  |  |
| Dairy-free cheese and spreads* | 2010 | 6 | 953 | 400 | 586 | 1143 | 1273 | 1316 |
|  | 2013 | 12 | 928 | 400 | 779 | 1017 | 1143^e^ | 1263 |
|  | 2017 | 12 | 825 | 167 | 758 | 800 | 975 | 1263 |
|  | 2020 | 35 | 770 | 304 | 709 | 800 | 896^e^ | 1526 |
|  |  |  |  |  |  |  |  |  |
| **Fats and Oils** | **2010** | **329** | **872** | **233** | **656^bc^** | **867^abc^** | **1000^bc^** | **1813** |
|  | **2013** | **360** | **814** | **12** | **644** | **800^ade^** | **1000^de^** | **2600** |
|  | **2017** | **410** | **727** | **19** | **600^b^** | **721^bd^** | **839^bd^** | **1728** |
|  | **2020** | **682** | **740** | **17** | **600^c^** | **741^ce^** | **858^ce^** | **1818** |
|  |  |  |  |  |  |  |  |  |
| Salted margarine | 2010 | 49 | 672 | 300 | 600 | 700^b^ | 700 | 1000 |
|  | 2013 | 49 | 653 | 300 | 600 | 650 | 700 | 850 |
|  | 2017 | 33 | 641 | 350 | 600 | 600^b^ | 700 | 1000 |
|  | 2020 | 82 | 624 | 350 | 563 | 650 | 700 | 900 |
|  |  |  |  |  |  |  |  |  |
| Salad dressing and mayonnaise | 2010 | 260 | 925 | 233 | 778^abc^ | 933^abc^ | 1067^bc^ | 1813 |
|  | 2013 | 292 | 851 | 12 | 667^a^ | 844^ade^ | 1000^de^ | 2600 |
|  | 2017 | 349 | 737 | 19 | 617^b^ | 738^bdf^ | 872^bd^ | 1728 |
|  | 2020 | 525 | 766 | 17 | 621^c^ | 774^cef^ | 891^ce^ | 1818 |
|  |  |  |  |  |  |  |  |  |
| Salted butter and dairy spreads | 2010 | 20 | 670 | 550 | 600 | 600^c^ | 763 | 950 |
|  | 2013 | 19 | 664 | 71 | 600 | 600^e^ | 775 | 950 |
|  | 2017 | 28 | 698 | 450 | 588 | 700 | 763 | 1200 |
|  | 2020 | 75 | 683 | 500 | 600 | 700^ce^ | 750 | 950 |
|  |  |  |  |  |  |  |  |  |
| **Fish and Seafood Products** | **2010** | **287** | **444** | **53** | **300** | **413^bc^** | **540^c^** | **1436** |
|  | **2013** | **397** | **540** | **27** | **291** | **388** | **536** | **8796** |
|  | **2017** | **378** | **504** | **32** | **280** | **377^b^** | **500** | **8796** |
|  | **2020** | **481** | **435** | **20** | **283** | **376^c^** | **500^c^** | **7600** |
|  |  |  |  |  |  |  |  |  |
| Canned tuna | 2010 | 28 | 312 | 83 | 250 | 309^c^ | 389 | 500 |
|  | 2013 | 47 | 281 | 27 | 241 | 288^de^ | 324^de^ | 596 |
|  | 2017 | 53 | 322 | 83 | 283 | 327^df^ | 392^df^ | 596 |
|  | 2020 | 84 | 389 | 91 | 287 | 392^cef^ | 468^ef^ | 748 |
|  |  |  |  |  |  |  |  |  |
| Imitation seafood* | 2010 | 7 | 602 | 553 | 556 | 588 | 646 | 670 |
|  | 2013 | 11 | 444 | 330 | 377 | 478 | 493 | 553 |
|  | 2017 | 7 | 419 | 340 | 340 | 450 | 468 | 529 |
|  | 2020 | 6 | 391 | 330 | 348 | 400 | 430 | 444 |
|  |  |  |  |  |  |  |  |  |
| Canned fish and seafood | 2010 | 109 | 390 | 80 | 298 | 400 | 473 | 774 |
|  | 2013 | 120 | 558 | 45 | 327^d^ | 400 | 536 | 6000 |
|  | 2017 | 113 | 525 | 45 | 273^d^ | 376 | 462 | 6000 |
|  | 2020 | 196 | 394 | 20 | 284 | 368 | 455 | 1091 |
|  |  |  |  |  |  |  |  |  |
| Frozen fish and seafood | 2010 | 143 | 503 | 53 | 313 | 455^c^ | 630^bc^ | 1436 |
|  | 2013 | 216 | 531 | 82 | 291 | 400 | 595 | 8796 |
|  | 2017 | 203 | 500 | 32 | 278 | 400 | 539^b^ | 8796 |
|  | 2020 | 191 | 417 | 48 | 280 | 376^c^ | 535^c^ | 1467 |
|  |  |  |  |  |  |  |  |  |
| Seafood spread and dips* | 2010 | 0 | --- | --- | --- | --- | --- | --- |
|  | 2013 | 3 | 4889 | 800 | 3533 | 6267 | 6933 | 7600 |
|  | 2017 | 2 | 4867 | 2133 | 3500 | 4867 | 6233 | 7600 |
|  | 2020 | 4 | 4356 | 768 | 1283 | 4527 | 7600 | 7600 |
|  |  |  |  |  |  |  |  |  |
| **Mixed Dishes** | **2010** | **1202** | **357** | **0** | **258^abc^** | **325** | **430** | **1214** |
|  | **2013** | **1515** | **353** | **6** | **244^a^** | **321** | **438^e^** | **1214** |
|  | **2017** | **1237** | **349** | **3** | **248^b^** | **324** | **431** | **1067** |
|  | **2020** | **1909** | **340** | **12** | **247^c^** | **316** | **417^e^** | **1182** |
|  |  |  |  |  |  |  |  |  |
| Canned and dry foods as consumed | 2010 | 360 | 331 | 0 | 263^abc^ | 310^abc^ | 378^bc^ | 822 |
|  | 2013 | 373 | 308 | 6 | 247^a^ | 283^a^ | 344 | 822 |
|  | 2017 | 234 | 311 | 3 | 249^b^ | 284^b^ | 339^b^ | 1018 |
|  | 2020 | 519 | 320 | 50 | 248^c^ | 292^c^ | 342^c^ | 822 |
|  |  |  |  |  |  |  |  |  |
| Refrigerated and frozen appetizers, sides and entrees | 2010 | 572 | 344 | 49 | 238 | 310 | 409^c^ | 1214 |
|  | 2013 | 778 | 348 | 18 | 231 | 317 | 421^e^ | 1214 |
|  | 2017 | 708 | 337 | 20 | 236 | 317 | 410^f^ | 1067 |
|  | 2020 | 893 | 328 | 104 | 236 | 310 | 387^cef^ | 1182 |
|  |  |  |  |  |  |  |  |  |
| Pizza, pizza snacks and frozen sandwiches | 2010 | 154 | 519 | 200 | 418 | 512^b^ | 594^bc^ | 906 |
|  | 2013 | 210 | 493 | 215 | 419 | 489 | 563 | 1000 |
|  | 2017 | 196 | 479 | 239 | 411 | 473^b^ | 547^b^ | 875 |
|  | 2020 | 246 | 489 | 271 | 421 | 489 | 550^c^ | 841 |
|  |  |  |  |  |  |  |  |  |
| Potatoes | 2010 | 75 | 270 | 18 | 200^c^ | 292 | 373 | 1178 |
|  | 2013 | 105 | 261 | 15 | 182^e^ | 294 | 353 | 595 |
|  | 2017 | 81 | 262 | 18 | 176^f^ | 259 | 353 | 541 |
|  | 2020 | 159 | 263 | 12 | 106^cef^ | 268 | 376 | 635 |
|  |  |  |  |  |  |  |  |  |
| Refrigerated prepared salads | 2010 | 41 | 317 | 104 | 250 | 300 | 365 | 1020 |
|  | 2013 | 49 | 359 | 105 | 237 | 313 | 440^e^ | 1136 |
|  | 2017 | 18 | 274 | 38 | 215 | 264 | 347 | 457 |
|  | 2020 | 92 | 293 | 15 | 226 | 295 | 360^e^ | 909 |
|  |  |  |  |  |  |  |  |  |
| **Meat and Meat Substitutes** | **2010** | **594** | **835** | **49** | **553^abc^** | **800^abc^** | **1000^b^** | **3000** |
|  | **2013** | **914** | **792** | **49** | **450^a^** | **705^ae^** | **960^d^** | **5000** |
|  | **2017** | **962** | **760** | **53** | **427^bf^** | **667^bf^** | **879^bdf^** | **5020** |
|  | **2020** | **1377** | **840** | **53** | **472^cf^** | **754^cef^** | **970^f^** | **3143** |
|  |  |  |  |  |  |  |  |  |
| Bacon and substitutes* | 2010 | 7 | 694 | 420 | 615 | 776 | 796 | 840 |
|  | 2013 | 37 | 642 | 298 | 560 | 617 | 759 | 1038 |
|  | 2017 | 43 | 680 | 260 | 496 | 655 | 755 | 2500 |
|  | 2020 | 57 | 631 | 259 | 500 | 611 | 761 | 1000 |
|  |  |  |  |  |  |  |  |  |
| Packaged sausages and wieners | 2010 | 100 | 912 | 245 | 797^bc^ | 872^bc^ | 1034^abc^ | 1857 |
|  | 2013 | 140 | 813 | 254 | 730 | 826 | 910^a^ | 1436 |
|  | 2017 | 140 | 806 | 480 | 718^b^ | 794^b^ | 868^b^ | 1429 |
|  | 2020 | 225 | 790 | 435 | 690^c^ | 792^c^ | 867^c^ | 1371 |
|  |  |  |  |  |  |  |  |  |
| Packaged deli meat | 2010 | 172 | 1092 | 424 | 885^abc^ | 1000^b^ | 1321^bc^ | 2429 |
|  | 2013 | 238 | 1086 | 394 | 811^a^ | 972^d^ | 1307^de^ | 2429 |
|  | 2017 | 243 | 1114 | 261 | 800^b^ | 886^bd^ | 1548^bd^ | 2400 |
|  | 2020 | 375 | 1149 | 480 | 818^c^ | 937 | 1517^ce^ | 2400 |
|  |  |  |  |  |  |  |  |  |
| Canned meat and poultry | 2010 | 46 | 770 | 241 | 602 | 805 | 960 | 1404 |
|  | 2013 | 21 | 848 | 280 | 750 | 867 | 960 | 1404 |
|  | 2017 | 27 | 753 | 274 | 645 | 780 | 901 | 1016 |
|  | 2020 | 56 | 788 | 238 | 655 | 833 | 929 | 1404 |
|  |  |  |  |  |  |  |  |  |
| Meat sticks and jerky | 2010 | 15 | 1759 | 750 | 1229 | 1733 | 2293^bc^ | 2771 |
|  | 2013 | 40 | 1595 | 750 | 1316^d^ | 1527^d^ | 1941 | 2533 |
|  | 2017 | 55 | 1366 | 750 | 1079^d^ | 1214^df^ | 1700^b^ | 2450 |
|  | 2020 | 117 | 1529 | 750 | 1218 | 1586^f^ | 1800^c^ | 2533 |
|  |  |  |  |  |  |  |  |  |
| Meat and poultry products | 2010 | 221 | 554 | 49 | 387^abc^ | 530^abc^ | 690^abc^ | 1330 |
|  | 2013 | 346 | 494 | 49 | 353^a^ | 449^ade^ | 562^ade^ | 5000 |
|  | 2017 | 376 | 448 | 53 | 330^bf^ | 408^bd^ | 520^bd^ | 5020 |
|  | 2020 | 395 | 447 | 53 | 354^cf^ | 421^ce^ | 522^ce^ | 1200 |
|  |  |  |  |  |  |  |  |  |
| Bacon bits and shelf stable pre-cooked bacon* | 2010 | 4 | 2531 | 1929 | 2411 | 2598 | 2719 | 3000 |
|  | 2013 | 19 | 1977 | 643 | 1286 | 2125 | 2500 | 3429 |
|  | 2017 | 8 | 2009 | 643 | 1277 | 2375 | 2714 | 2714 |
|  | 2020 | 19 | 2044 | 889 | 1518 | 2143 | 2536 | 3143 |
|  |  |  |  |  |  |  |  |  |
| Meat substitutes | 2010 | 29 | 610 | 135 | 460 | 600^ac^ | 774^ac^ | 1154 |
|  | 2013 | 73 | 516 | 282 | 400 | 471^a^ | 547^a^ | 1020 |
|  | 2017 | 70 | 541 | 282 | 415 | 474 | 676 | 1154 |
|  | 2020 | 133 | 558 | 135 | 386 | 451^c^ | 632^c^ | 1929 |
|  |  |  |  |  |  |  |  |  |
| **Soups** | **2010** | **333** | **270** | **33** | **224^ab^** | **260^a^** | **318** | **628** |
|  | **2013** | **463** | **250** | **5** | **191^ade^** | **248^ae^** | **300^e^** | **536** |
|  | **2017** | **491** | **253** | **2** | **203^bd^** | **249^f^** | **304^f^** | **632** |
|  | **2020** | **865** | **261** | **0** | **211^e^** | **260^ef^** | **316^ef^** | **943** |
|  |  |  |  |  |  |  |  |  |
| Bouillon and broth products as consumed | 2010 | 47 | 285 | 33 | 237^c^ | 276 | 360^b^ | 469 |
|  | 2013 | 110 | 264 | 16 | 213 | 264 | 350^d^ | 494 |
|  | 2017 | 125 | 242 | 2 | 202 | 242 | 311^bdf^ | 494 |
|  | 2020 | 205 | 248 | 0 | 185^c^ | 243 | 349^f^ | 943 |
|  |  |  |  |  |  |  |  |  |
| Canned condensed wet soup as consumed | 2010 | 71 | 291 | 128 | 248^abc^ | 291^a^ | 332^ac^ | 477 |
|  | 2013 | 76 | 251 | 128 | 200^a^ | 248^a^ | 298^a^ | 399 |
|  | 2017 | 72 | 257 | 8 | 199^b^ | 267 | 307 | 380 |
|  | 2020 | 125 | 251 | 9 | 183^c^ | 266 | 303^c^ | 498 |
|  |  |  |  |  |  |  |  |  |
| Ready-to-serve soup | 2010 | 142 | 247 | 55 | 189^c^ | 252 | 281^a^ | 474 |
|  | 2013 | 147 | 229 | 8 | 188^de^ | 234 | 256^ade^ | 536 |
|  | 2017 | 157 | 249 | 99 | 209^d^ | 250 | 292^d^ | 560 |
|  | 2020 | 254 | 252 | 49 | 223^ce^ | 256 | 294^e^ | 430 |
|  |  |  |  |  |  |  |  |  |
| Fresh and instant oriental noodle soups as consumed | 2010 | 17 | 222 | 37 | 154^ac^ | 183^c^ | 336 | 451 |
|  | 2013 | 76 | 258 | 110 | 204^a^ | 236^e^ | 313 | 451 |
|  | 2017 | 93 | 261 | 129 | 199 | 236^f^ | 312 | 632 |
|  | 2020 | 202 | 284 | 102 | 207^c^ | 286^cef^ | 328 | 632 |
|  |  |  |  |  |  |  |  |  |
| Dry soup mixes as consumed | 2010 | 56 | 305 | 135 | 252 | 280 | 345 | 628 |
|  | 2013 | 54 | 268 | 5 | 243 | 260 | 306 | 463 |
|  | 2017 | 44 | 272 | 146 | 232 | 253 | 334 | 395 |
|  | 2020 | 79 | 279 | 0 | 249 | 273 | 321 | 428 |
|  |  |  |  |  |  |  |  |  |
| **Snacks** | **2010** | **454** | **580** | **0** | **300** | **571^abc^** | **779^abc^** | **2080** |
|  | **2013** | **722** | **522** | **0** | **270** | **500^a^** | **720^a^** | **2156** |
|  | **2017** | **829** | **505** | **0** | **267** | **480^b^** | **697^b^** | **2020** |
|  | **2020** | **1257** | **517** | **0** | **280** | **500^c^** | **700^c^** | **1933** |
|  |  |  |  |  |  |  |  |  |
| Snack foods | 2010 | 336 | 698 | 0 | 516^bc^ | 678^abc^ | 852^bc^ | 2080 |
|  | 2013 | 459 | 653 | 0 | 460 | 620^a^ | 810^e^ | 1750 |
|  | 2017 | 531 | 621 | 0 | 420^b^ | 600^b^ | 780^b^ | 1800 |
|  | 2020 | 914 | 610 | 0 | 440^c^ | 600^c^ | 760^ce^ | 1920 |
|  |  |  |  |  |  |  |  |  |
| Salted snacking fruits, nuts and seeds | 2010 | 64 | 308 | 10 | 148 | 275 | 405 | 1340 |
|  | 2013 | 159 | 389 | 0 | 190 | 311 | 500 | 2156 |
|  | 2017 | 207 | 367 | 18 | 190 | 320 | 500 | 2020 |
|  | 2020 | 222 | 343 | 0 | 148 | 300 | 500 | 1933 |
|  |  |  |  |  |  |  |  |  |
| Pudding as consumed | 2010 | 54 | 170 | 39 | 124 | 146 | 231^c^ | 312 |
|  | 2013 | 104 | 149 | 25 | 111 | 136 | 202^e^ | 312 |
|  | 2017 | 91 | 147 | 12 | 106 | 126 | 188 | 312 |
|  | 2020 | 121 | 138 | 12 | 111 | 126 | 152^ce^ | 313 |
|  |  |  |  |  |  |  |  |  |
| **Sauces, Dips, Gravies, and Condiments** | **2010** | **683** | **833** | **0** | **393^ab^** | **575^ab^** | **967** | **5800** |
|  | **2013** | **1068** | **910** | **0** | **367^a^** | **527^a^** | **938^e^** | **9600** |
|  | **2017** | **1154** | **931** | **0** | **360^bf^** | **504^bf^** | **1009** | **9333** |
|  | **2020** | **1640** | **1032** | **0** | **383^f^** | **550^f^** | **1055^e^** | **10214** |
|  |  |  |  |  |  |  |  |  |
| Tomato sauce | 2010 | 13 | 256 | 15 | 38 | 308 | 385 | 485 |
|  | 2013 | 17 | 297 | 15 | 231 | 308 | 385 | 736 |
|  | 2017 | 16 | 277 | 8 | 232 | 314 | 390 | 448 |
|  | 2020 | 50 | 259 | 12 | 162 | 247 | 379 | 449 |
|  |  |  |  |  |  |  |  |  |
| Other sauces, dips, gravies, and condiments | 2010 | 502 | 983 | 0 | 467^ab^ | 733^ab^ | 1200 | 5800 |
|  | 2013 | 823 | 1055 | 0 | 419^ae^ | 633^a^ | 1123 | 9600 |
|  | 2017 | 911 | 1080 | 0 | 405^bf^ | 627^b^ | 1226 | 9333 |
|  | 2020 | 1309 | 1195 | 0 | 444^ef^ | 673 | 1224 | 10214 |
|  |  |  |  |  |  |  |  |  |
| Pasta sauce | 2010 | 168 | 431 | 136 | 330^abc^ | 397^bc^ | 496^bc^ | 1267 |
|  | 2013 | 228 | 430 | 70 | 292^a^ | 372^d^ | 468^d^ | 3600 |
|  | 2017 | 227 | 379 | 48 | 286^b^ | 344^bd^ | 424^bd^ | 1400 |
|  | 2020 | 281 | 413 | 91 | 298^c^ | 355^c^ | 448^c^ | 1400 |
|  |  |  |  |  |  |  |  |  |
| **Canned and Bottled Vegetables and Legumes**^†^ | **2010** | **528** | **499** | **0** | **222^abc^** | **311^b^** | **588^abc^** | **2800** |
|  | **2013** | **665** | **541** | **0** | **178^ade^** | **293^d^** | **800^a^** | **3500** |
|  | **2017** | **617** | **530** | **0** | **99^bdf^** | **244^bdf^** | **800^b^** | **4667** |
|  | **2020** | **972** | **605** | **0** | **146^cef^** | **328^f^** | **900^c^** | **4333** |
|  |  |  |  |  |  |  |  |  |
| **Seasoning Mixes** | **2010** | **37** | **4529** | **33** | **1286** | **5250** | **6452^bc^** | **10741** |
|  | **2013** | **191** | **5913** | **0** | **2462** | **5333** | **7829^de^** | **30000** |
|  | **2017** | **203** | **7148** | **0** | **3042** | **6286** | **9512^bd^** | **30000** |
|  | **2020** | **392** | **7385** | **0** | **2667** | **6000** | **10292^ce^** | **30000** |
|  |  |  |  |  |  |  |  |  |
| Breading, batter and coating mixes* | 2010 | 2 | 3542 | 3500 | 3521 | 3542 | 3563 | 3583 |
|  | 2013 | 33 | 2567 | 500 | 2077 | 2385 | 3333 | 4167 |
|  | 2017 | 14 | 2952 | 250 | 2517 | 2833 | 3271 | 5500 |
|  | 2020 | 43 | 2587 | 250 | 2000 | 2667 | 3600 | 3933 |
|  |  |  |  |  |  |  |  |  |
| Seasoning | 2010 | 35 | 4586 | 33 | 943^abc^ | 5333^c^ | 6781^bc^ | 10741 |
|  | 2013 | 158 | 6612 | 0 | 3554^a^ | 6145 | 8371^de^ | 30000 |
|  | 2017 | 189 | 7459 | 0 | 3500^b^ | 6889 | 10000^bd^ | 30000 |
|  | 2020 | 349 | 7976 | 0 | 4000^c^ | 7000^c^ | 11000^ce^ | 30000 |
|  |  |  |  |  |  |  |  |  |
| **Infant and Toddler Foods**^‡^ | **2017** | **106** | **92** | **0** | **4** | **28^f^** | **143** | **500** |
|  | **2020** | **233** | **104** | **0** | **19** | **78^f^** | **125** | **500** |
|  |  |  |  |  |  |  |  |  |
| Cookies, biscuits and snack bars | 2017 | 39 | 96 | 0 | 0 | 21 | 190 | 357 |
|  | 2020 | 103 | 102 | 0 | 0 | 39 | 197 | 380 |
|  |  |  |  |  |  |  |  |  |
| Mixed dishes | 2017 | 55 | 59 | 0 | 7^f^ | 25^f^ | 88 | 314 |
|  | 2020 | 90 | 85 | 0 | 72^f^ | 82^f^ | 90 | 200 |
|  |  |  |  |  |  |  |  |  |
| Savoury snacks | 2017 | 12 | 234 | 10 | 66 | 182 | 500^f^ | 500 |
|  | 2020 | 40 | 151 | 0 | 21 | 71 | 214^f^ | 500 |
|  |  |  |  |  |  |  |  |  |
| **Salted Nut Butters**^†^ | **2010** | **30** | **273** | **0** | **102^ac^** | **333** | **400** | **467** |
|  | **2013** | **35** | **343** | **109** | **300^ad^** | **357** | **433** | **667** |
|  | **2017** | **51** | **300** | **109** | **222^df^** | **300^f^** | **371** | **533** |
|  | **2020** | **78** | **344** | **67** | **303^cf^** | **367^f^** | **400** | **500** |

**Note**: Pairwise comparisons between the 25^th^, 50^th^, and 75^th^ percentile sodium content for each year-pair (i.e., 2010-2013, 2010-2017, 2010-2020, 2013-2017, 2013-2020 and 2017-2020) were made using quantile regression models. Analysis by major category only is provided in Table 1.

*Models were not fit for pairwise comparisons with n<10 in either year.

^†^There is only one subcategory under the canned and bottled vegetables and legumes and salted nut butters major categories.

^‡^Infant and Toddler Foods were not collected in the FLIP 2010 and 2013 collections.

^a^p<0.05 for comparison between 2010-2013 from quantile regression.

^b^p<0.05 for comparison between 2010-2017 from quantile regression.

^c^p<0.05 for comparison between 2010-2020 from quantile regression.

^d^p<0.05 for comparison between 2013-2017 from quantile regression.

^e^p<0.05 for comparison between 2013-2020 from quantile regression.

^f^p<0.05 for comparison between 2017-2020 from quantile regression.

**Supplementary Table 3.** Proportion of processed foods meeting the average sodium reduction targets and exceeding the maximum sodium levels set by Health Canada for 2012-2016* in 2010, 2013, 2017, and 2020 by major category and subcategory.

|  |  |  | **Average Target** | |  | **Maximum Level** | |
| --- | --- | --- | --- | --- | --- | --- | --- |
|  | **Year** | **n** | **% Meeting Average Target** | **p-trend** |  | **% Exceeding Maximum Level** | **p-trend** |
| Bakery Products | 2010 | 1768 | 30^bc^ | <0.001 |  | 23^abc^ | <0.001 |
|  | 2013 | 2222 | 31^e^ |  |  | 18^a^ |  |
|  | 2017 | 2935 | 33^b^ |  |  | 16^b^ |  |
|  | 2020 | 4097 | 34^ce^ |  |  | 17^c^ |  |
|  |  |  |  |  |  |  |  |
| Bread products | 2010 | 379 | 21 | 0.413 |  | 21^a^ | 0.009 |
|  | 2013 | 444 | 26^e^ |  |  | 14^ade^ |  |
|  | 2017 | 656 | 24 |  |  | 23^d^ |  |
|  | 2020 | 674 | 20^e^ |  |  | 24^e^ |  |
|  |  |  |  |  |  |  |  |
| Cookies | 2010 | 309 | 38 | 0.666 |  | 27^abc^ | <0.001 |
|  | 2013 | 397 | 40 |  |  | 20^ade^ |  |
|  | 2017 | 543 | 36 |  |  | 15^bd^ |  |
|  | 2020 | 844 | 38 |  |  | 15^ce^ |  |
|  |  |  |  |  |  |  |  |
| Toaster pastries | 2010 | 12 | 33 | 0.333 |  | 17 | 0.398 |
|  | 2013 | 11 | 55 |  |  | 9 |  |
|  | 2017 | 11 | 27 |  |  | 18 |  |
|  | 2020 | 39 | 54 |  |  | 23 |  |
|  |  |  |  |  |  |  |  |
| Tortillas and wraps | 2010 | 27 | 30^b^ | <0.001 |  | 15 | 0.138 |
|  | 2013 | 63 | 21^de^ |  |  | 16 |  |
|  | 2017 | 52 | 62^bd^ |  |  | 4 |  |
|  | 2020 | 86 | 49^e^ |  |  | 9 |  |
|  |  |  |  |  |  |  |  |
| Granola bars | 2010 | 172 | 22^bc^ | <0.001 |  | 20 | 0.815 |
|  | 2013 | 199 | 24^de^ |  |  | 13 |  |
|  | 2017 | 379 | 36^bd^ |  |  | 18 |  |
|  | 2020 | 792 | 34^ce^ |  |  | 18 |  |
|  |  |  |  |  |  |  |  |
| Dough and pastry | 2010 | 29 | 10 | 0.111 |  | 34^ac^ | 0.104 |
|  | 2013 | 47 | 21 |  |  | 13^a^ |  |
|  | 2017 | 59 | 14 |  |  | 20 |  |
|  | 2020 | 73 | 27 |  |  | 14^c^ |  |
|  |  |  |  |  |  |  |  |
| Crackers | 2010 | 268 | 42 | 0.536 |  | 28^bc^ | <0.001 |
|  | 2013 | 301 | 36^d^ |  |  | 23^d^ |  |
|  | 2017 | 317 | 44^d^ |  |  | 16^bd^ |  |
|  | 2020 | 553 | 41 |  |  | 18^c^ |  |
|  |  |  |  |  |  |  |  |
| Dry bread | 2010 | 65 | 25 | 0.704 |  | 28 | 0.236 |
|  | 2013 | 107 | 25 |  |  | 23 |  |
|  | 2017 | 117 | 32 |  |  | 19 |  |
|  | 2020 | 173 | 22 |  |  | 21 |  |
|  |  |  |  |  |  |  |  |
| Breadcrumbs, croutons and salad toppers | 2010 | 56 | 21^bc^ | <0.001 |  | 16 | 0.122 |
|  | 2013 | 64 | 28^e^ |  |  | 25^e^ |  |
|  | 2017 | 88 | 42^b^ |  |  | 14 |  |
|  | 2020 | 95 | 47^ce^ |  |  | 12^e^ |  |
|  |  |  |  |  |  |  |  |
| Commercial and frozen baked desserts | 2010 | 383 | 33 | 0.719 |  | 21^bc^ | <0.001 |
|  | 2013 | 478 | 32 |  |  | 17^d^ |  |
|  | 2017 | 613 | 32 |  |  | 10^bdf^ |  |
|  | 2020 | 615 | 34 |  |  | 15^cf^ |  |
|  |  |  |  |  |  |  |  |
| Breakfast foods (e.g., pancakes, waffles, scones) | 2010 | 68 | 13^abc^ | 0.002 |  | 19^bc^ | <0.001 |
|  | 2013 | 111 | 32^a^ |  |  | 13^e^ |  |
|  | 2017 | 100 | 34^b^ |  |  | 6^b^ |  |
|  | 2020 | 153 | 37^c^ |  |  | 5^ce^ |  |
|  |  |  |  |  |  |  |  |
| Breakfast Cereals | 2010 | 281 | 42^ab^ | 0.210 |  | 15^abc^ | <0.001 |
|  | 2013 | 309 | 51^ad^ |  |  | 9^ad^ |  |
|  | 2017 | 339 | 63^bdf^ |  |  | 5^bd^ |  |
|  | 2020 | 755 | 48^f^ |  |  | 6^c^ |  |
|  |  |  |  |  |  |  |  |
| Ready-to-eat-cereals | 2010 | 230 | 45^ab^ | 0.445 |  | 15^bc^ | <0.001 |
|  | 2013 | 250 | 56^ae^ |  |  | 10 |  |
|  | 2017 | 256 | 62^bf^ |  |  | 6^b^ |  |
|  | 2020 | 651 | 46^ef^ |  |  | 7^c^ |  |
|  |  |  |  |  |  |  |  |
| Hot instant cereals | 2010 | 51 | 29^bc^ | <0.001 |  | 14^bc^ | <0.001 |
|  | 2013 | 59 | 29^de^ |  |  | 3 |  |
|  | 2017 | 83 | 65^bd^ |  |  | 0^b^ |  |
|  | 2020 | 104 | 60^ce^ |  |  | 0^c^ |  |
|  |  |  |  |  |  |  |  |
| Dairy Products and Substitutes | 2010 | 403 | 50 | 0.085 |  | 15 | 0.102 |
|  | 2013 | 505 | 49 |  |  | 13 |  |
|  | 2017 | 612 | 47 |  |  | 12 |  |
|  | 2020 | 1059 | 46 |  |  | 11 |  |
|  |  |  |  |  |  |  |  |
| Cheese | 2010 | 348 | 55^c^ | 0.003 |  | 12 | 0.679 |
|  | 2013 | 437 | 53^e^ |  |  | 10 |  |
|  | 2017 | 540 | 49 |  |  | 11 |  |
|  | 2020 | 945 | 47^ce^ |  |  | 12 |  |
|  |  |  |  |  |  |  |  |
| Processed cheese and other cheese products | 2010 | 49 | 20^c^ | <0.001 |  | 24^c^ | 0.004 |
|  | 2013 | 56 | 21^e^ |  |  | 32^e^ |  |
|  | 2017 | 60 | 30^f^ |  |  | 25^f^ |  |
|  | 2020 | 79 | 47^cef^ |  |  | 8^cef^ |  |
|  |  |  |  |  |  |  |  |
| Dairy-free cheese and spreads^†^ | 2010 | 6 | 33 | n/a |  | 67 | n/a |
|  | 2013 | 12 | 17 |  |  | 33^e^ |  |
|  | 2017 | 12 | 8 |  |  | 8 |  |
|  | 2020 | 35 | 14 |  |  | 3^e^ |  |
|  |  |  |  |  |  |  |  |
| Fats and Oils | 2010 | 329 | 14^b^ | 0.129 |  | 22^b^ | 0.004 |
|  | 2013 | 360 | 14^d^ |  |  | 25^de^ |  |
|  | 2017 | 410 | 20^bd^ |  |  | 15^bd^ |  |
|  | 2020 | 682 | 16 |  |  | 17^e^ |  |
|  |  |  |  |  |  |  |  |
| Salted margarine | 2010 | 49 | 2 | 0.170 |  | 6 | 0.762 |
|  | 2013 | 49 | 2 |  |  | 2 |  |
|  | 2017 | 33 | 6 |  |  | 6 |  |
|  | 2020 | 82 | 6 |  |  | 4 |  |
|  |  |  |  |  |  |  |  |
| Salad dressing and mayonnaise | 2010 | 260 | 17 | 0.110 |  | 25^b^ | 0.005 |
|  | 2013 | 292 | 17 |  |  | 30^de^ |  |
|  | 2017 | 349 | 23 |  |  | 16^bd^ |  |
|  | 2020 | 525 | 20 |  |  | 21^e^ |  |
|  |  |  |  |  |  |  |  |
| Salted butter and dairy spreads | 2010 | 20 | 0 | 0.429 |  | 10 | 0.382 |
|  | 2013 | 19 | 5 |  |  | 11 |  |
|  | 2017 | 28 | 4 |  |  | 14 |  |
|  | 2020 | 75 | 0 |  |  | 5 |  |
|  |  |  |  |  |  |  |  |
| Fish and Seafood Products | 2010 | 287 | 29 | 0.497 |  | 20^b^ | 0.054 |
|  | 2013 | 397 | 30 |  |  | 19^d^ |  |
|  | 2017 | 378 | 31 |  |  | 12^bd^ |  |
|  | 2020 | 481 | 31 |  |  | 17 |  |
|  |  |  |  |  |  |  |  |
| Canned tuna | 2010 | 28 | 29^c^ | 0.003 |  | 21 | 0.008 |
|  | 2013 | 47 | 34^e^ |  |  | 6^e^ |  |
|  | 2017 | 53 | 23 |  |  | 11^f^ |  |
|  | 2020 | 84 | 11^ce^ |  |  | 33^ef^ |  |
|  |  |  |  |  |  |  |  |
| Imitation seafood^†^ | 2010 | 7 | 0 | n/a |  | 0 | n/a |
|  | 2013 | 11 | 91 |  |  | 0 |  |
|  | 2017 | 7 | 100 |  |  | 0 |  |
|  | 2020 | 6 | 100 |  |  | 0 |  |
|  |  |  |  |  |  |  |  |
| Canned fish and seafood | 2010 | 109 | 34 | 0.534 |  | 14 | 0.945 |
|  | 2013 | 120 | 27 |  |  | 18 |  |
|  | 2017 | 113 | 37 |  |  | 14 |  |
|  | 2020 | 196 | 34 |  |  | 16 |  |
|  |  |  |  |  |  |  |  |
| Frozen fish and seafood | 2010 | 143 | 27 | 0.080 |  | 26^bc^ | <0.001 |
|  | 2013 | 216 | 28 |  |  | 22^de^ |  |
|  | 2017 | 203 | 29 |  |  | 10^bd^ |  |
|  | 2020 | 191 | 36 |  |  | 9^ce^ |  |
|  |  |  |  |  |  |  |  |
| Seafood spread and dips^†^ | 2010 | 0 | --- | n/a |  | --- | n/a |
|  | 2013 | 3 | 0 |  |  | 100 |  |
|  | 2017 | 2 | 0 |  |  | 100 |  |
|  | 2020 | 4 | 0 |  |  | 100 |  |
|  |  |  |  |  |  |  |  |
| Mixed Dishes | 2010 | 1202 | 28^a^ | 0.494 |  | 18^bc^ | 0.007 |
|  | 2013 | 1515 | 34^a^ |  |  | 17 |  |
|  | 2017 | 1237 | 31 |  |  | 15^b^ |  |
|  | 2020 | 1909 | 31 |  |  | 15^c^ |  |
|  |  |  |  |  |  |  |  |
| Canned and dry foods as consumed | 2010 | 360 | 30^ab^ | 0.232 |  | 13 | 0.885 |
|  | 2013 | 373 | 42^a^ |  |  | 12 |  |
|  | 2017 | 234 | 43^b^ |  |  | 8 |  |
|  | 2020 | 519 | 36 |  |  | 13 |  |
|  |  |  |  |  |  |  |  |
| Refrigerated and frozen appetizers, sides and entrees | 2010 | 572 | 29^a^ | 0.687 |  | 22^c^ | 0.042 |
|  | 2013 | 778 | 35^a^ |  |  | 20 |  |
|  | 2017 | 708 | 30 |  |  | 19 |  |
|  | 2020 | 893 | 32 |  |  | 18^c^ |  |
|  |  |  |  |  |  |  |  |
| Pizza, pizza snacks and frozen sandwiches | 2010 | 154 | 19 | 0.240 |  | 21^abc^ | <0.001 |
|  | 2013 | 210 | 19 |  |  | 13^ade^ |  |
|  | 2017 | 196 | 20 |  |  | 7^bd^ |  |
|  | 2020 | 246 | 15 |  |  | 6^ce^ |  |
|  |  |  |  |  |  |  |  |
| Potatoes | 2010 | 75 | 36 | 0.713 |  | 13 | 0.022 |
|  | 2013 | 105 | 32 |  |  | 13 |  |
|  | 2017 | 81 | 36 |  |  | 19 |  |
|  | 2020 | 159 | 36 |  |  | 23 |  |
|  |  |  |  |  |  |  |  |
| Refrigerated prepared salads | 2010 | 41 | 20 | 0.476 |  | 17 | 0.111 |
|  | 2013 | 49 | 22 |  |  | 31^e^ |  |
|  | 2017 | 18 | 28 |  |  | 11 |  |
|  | 2020 | 92 | 25 |  |  | 13^e^ |  |
|  |  |  |  |  |  |  |  |
| Meat and Meat Substitutes | 2010 | 594 | 30^abc^ | <0.001 |  | 61^abc^ | <0.001 |
|  | 2013 | 914 | 40^ad^ |  |  | 46^ade^ |  |
|  | 2017 | 962 | 48^bdf^ |  |  | 39^bd^ |  |
|  | 2020 | 1377 | 43^cf^ |  |  | 41^ce^ |  |
|  |  |  |  |  |  |  |  |
| Bacon and substitutes^†^ | 2010 | 7 | 29 | n/a |  | 71 | n/a |
|  | 2013 | 37 | 41 |  |  | 51^e^ |  |
|  | 2017 | 43 | 42 |  |  | 53^f^ |  |
|  | 2020 | 57 | 58 |  |  | 30^ef^ |  |
|  |  |  |  |  |  |  |  |
| Packaged sausages and wieners | 2010 | 100 | 18^abc^ | <0.001 |  | 65^abc^ | <0.001 |
|  | 2013 | 140 | 41^a^ |  |  | 42^ae^ |  |
|  | 2017 | 140 | 47^b^ |  |  | 34^b^ |  |
|  | 2020 | 225 | 47^c^ |  |  | 31^ce^ |  |
|  |  |  |  |  |  |  |  |
| Packaged deli meat | 2010 | 172 | 31^b^ | 0.794 |  | 63^bc^ | 0.012 |
|  | 2013 | 238 | 36 |  |  | 57 |  |
|  | 2017 | 243 | 42^bf^ |  |  | 49^b^ |  |
|  | 2020 | 375 | 33^f^ |  |  | 53^c^ |  |
|  |  |  |  |  |  |  |  |
| Canned meat and poultry | 2010 | 46 | 30 | 0.188 |  | 65 | 0.397 |
|  | 2013 | 21 | 14^d^ |  |  | 76 |  |
|  | 2017 | 27 | 44^d^ |  |  | 56 |  |
|  | 2020 | 56 | 38 |  |  | 61 |  |
|  |  |  |  |  |  |  |  |
| Meat sticks and jerky | 2010 | 15 | 27 | 0.187 |  | 73 | 0.244 |
|  | 2013 | 40 | 33 |  |  | 60 |  |
|  | 2017 | 55 | 49 |  |  | 47 |  |
|  | 2020 | 117 | 42 |  |  | 55 |  |
|  |  |  |  |  |  |  |  |
| Meat and poultry products | 2010 | 221 | 38^abc^ | <0.001 |  | 57^abc^ | <0.001 |
|  | 2013 | 346 | 50^ad^ |  |  | 44^ade^ |  |
|  | 2017 | 376 | 60^bd^ |  |  | 33^bd^ |  |
|  | 2020 | 395 | 57^c^ |  |  | 35^ce^ |  |
|  |  |  |  |  |  |  |  |
| Bacon bits and shelf stable pre-cooked bacon^†^ | 2010 | 4 | 0 | n/a |  | 0 | n/a |
|  | 2013 | 19 | 37 |  |  | 5 |  |
|  | 2017 | 8 | 38 |  |  | 0 |  |
|  | 2020 | 19 | 32 |  |  | 5 |  |
|  |  |  |  |  |  |  |  |
| Meat substitutes | 2010 | 29 | 17 | 0.105 |  | 59^abc^ | 0.363 |
|  | 2013 | 73 | 14 |  |  | 23^a^ |  |
|  | 2017 | 70 | 9^f^ |  |  | 34^b^ |  |
|  | 2020 | 133 | 24^f^ |  |  | 32^c^ |  |
|  |  |  |  |  |  |  |  |
| Soups | 2010 | 333 | 29^abc^ | 0.486 |  | 12 | 0.549 |
|  | 2013 | 463 | 44^ae^ |  |  | 11 |  |
|  | 2017 | 491 | 44^bf^ |  |  | 8 |  |
|  | 2020 | 865 | 36^cef^ |  |  | 11 |  |
|  |  |  |  |  |  |  |  |
| Bouillon and broth products as consumed | 2010 | 47 | 28^bc^ | 0.012 |  | 26^b^ | 0.793 |
|  | 2013 | 110 | 39 |  |  | 21^d^ |  |
|  | 2017 | 125 | 49^b^ |  |  | 11^bdf^ |  |
|  | 2020 | 205 | 47^c^ |  |  | 22^f^ |  |
|  |  |  |  |  |  |  |  |
| Canned condensed wet soup as consumed | 2010 | 71 | 14^abc^ | 0.048 |  | 8 | 0.132 |
|  | 2013 | 76 | 41^a^ |  |  | 1 |  |
|  | 2017 | 72 | 36^b^ |  |  | 4 |  |
|  | 2020 | 125 | 34^c^ |  |  | 2 |  |
|  |  |  |  |  |  |  |  |
| Ready-to-serve soup | 2010 | 142 | 38^a^ | 0.023 |  | 6 | 0.051 |
|  | 2013 | 147 | 53^ae^ |  |  | 6 |  |
|  | 2017 | 157 | 43^f^ |  |  | 4 |  |
|  | 2020 | 254 | 32^ef^ |  |  | 2 |  |
|  |  |  |  |  |  |  |  |
| Fresh and instant oriental noodle soups as consumed | 2010 | 17 | 65^c^ | 0.004 |  | 6 | 0.229 |
|  | 2013 | 76 | 53^e^ |  |  | 12 |  |
|  | 2017 | 93 | 51^f^ |  |  | 9 |  |
|  | 2020 | 202 | 38^cef^ |  |  | 15 |  |
|  |  |  |  |  |  |  |  |
| Dry soup mixes as consumed | 2010 | 56 | 13^b^ | 0.520 |  | 21 | 0.096 |
|  | 2013 | 54 | 24 |  |  | 15 |  |
|  | 2017 | 44 | 36^bf^ |  |  | 16 |  |
|  | 2020 | 79 | 16^f^ |  |  | 10 |  |
|  |  |  |  |  |  |  |  |
| Snacks | 2010 | 454 | 22^abc^ | 0.100 |  | 22^bc^ | <0.001 |
|  | 2013 | 722 | 31^a^ |  |  | 18^e^ |  |
|  | 2017 | 829 | 31^b^ |  |  | 16^b^ |  |
|  | 2020 | 1257 | 28^c^ |  |  | 13^ce^ |  |
|  |  |  |  |  |  |  |  |
| Snack foods | 2010 | 336 | 15^abc^ | 0.016 |  | 19^abc^ | <0.001 |
|  | 2013 | 459 | 23^a^ |  |  | 13^ae^ |  |
|  | 2017 | 531 | 26^b^ |  |  | 11^b^ |  |
|  | 2020 | 914 | 23^c^ |  |  | 10^ce^ |  |
|  |  |  |  |  |  |  |  |
| Salted snacking fruits, nuts and seeds | 2010 | 64 | 66 | 0.091 |  | 16 | 0.650 |
|  | 2013 | 159 | 60 |  |  | 20 |  |
|  | 2017 | 207 | 53 |  |  | 19 |  |
|  | 2020 | 222 | 55 |  |  | 16 |  |
|  |  |  |  |  |  |  |  |
| Pudding as consumed | 2010 | 54 | 7 | 0.375 |  | 50^c^ | 0.022 |
|  | 2013 | 104 | 18 |  |  | 37 |  |
|  | 2017 | 91 | 15 |  |  | 36 |  |
|  | 2020 | 121 | 17 |  |  | 30^c^ |  |
|  |  |  |  |  |  |  |  |
| Sauces, Dips, Gravies and Condiments | 2010 | 683 | 25^abc^ | <0.001 |  | 23^b^ | 0.542 |
|  | 2013 | 1068 | 31^ad^ |  |  | 19^d^ |  |
|  | 2017 | 1154 | 37^bdf^ |  |  | 16^bdf^ |  |
|  | 2020 | 1640 | 32^cf^ |  |  | 21^f^ |  |
|  |  |  |  |  |  |  |  |
| Tomato sauce | 2010 | 13 | 31 | 0.102 |  | 0 | 0.296 |
|  | 2013 | 17 | 35 |  |  | 6 |  |
|  | 2017 | 16 | 38 |  |  | 0 |  |
|  | 2020 | 50 | 52 |  |  | 0 |  |
|  |  |  |  |  |  |  |  |
| Other sauces, dips, gravies, and condiments | 2010 | 502 | 25^abc^ | 0.049 |  | 25^ab^ | 0.879 |
|  | 2013 | 823 | 31^ad^ |  |  | 21^ae^ |  |
|  | 2017 | 911 | 36^bdf^ |  |  | 18^bf^ |  |
|  | 2020 | 1309 | 30^cf^ |  |  | 24^ef^ |  |
|  |  |  |  |  |  |  |  |
| Pasta sauce | 2010 | 168 | 26^bc^ | <0.001 |  | 16^bc^ | 0.005 |
|  | 2013 | 228 | 32^d^ |  |  | 14 |  |
|  | 2017 | 227 | 43^bd^ |  |  | 8^b^ |  |
|  | 2020 | 281 | 40^c^ |  |  | 9^c^ |  |
|  |  |  |  |  |  |  |  |
| Canned and Bottled Vegetables and Legumes^‡^ | 2010 | 528 | 16^abc^ | <0.001 |  | 29^abc^ | <0.001 |
|  | 2013 | 665 | 25^ade^ |  |  | 20^ade^ |  |
|  | 2017 | 617 | 39^bdf^ |  |  | 14^bd^ |  |
|  | 2020 | 972 | 32^cef^ |  |  | 15^ce^ |  |
|  |  |  |  |  |  |  |  |
| Seasoning Mixes | 2010 | 37 | 41 | 0.289 |  | 8 | 0.019 |
|  | 2013 | 191 | 39 |  |  | 17 |  |
|  | 2017 | 203 | 48^f^ |  |  | 17 |  |
|  | 2020 | 392 | 35^f^ |  |  | 23 |  |
|  |  |  |  |  |  |  |  |
| Breading, batter and coating mixes^†^ | 2010 | 2 | 0 | n/a |  | 100 | n/a |
|  | 2013 | 33 | 15 |  |  | 21 |  |
|  | 2017 | 14 | 7 |  |  | 14 |  |
|  | 2020 | 43 | 12 |  |  | 28 |  |
|  |  |  |  |  |  |  |  |
| Seasoning | 2010 | 35 | 43 | 0.195 |  | 3^c^ | 0.008 |
|  | 2013 | 158 | 44 |  |  | 16 |  |
|  | 2017 | 189 | 51^f^ |  |  | 17 |  |
|  | 2020 | 349 | 38^f^ |  |  | 22^c^ |  |
|  |  |  |  |  |  |  |  |
| Infant and Toddler Foods^§^ | 2017 | 106 | 81 | 0.373 |  | 6 | 0.339 |
|  | 2020 | 233 | 85 |  |  | 3 |  |
|  |  |  |  |  |  |  |  |
| Cookies, biscuits and snack bars | 2017 | 39 | 72 | 0.903 |  | 8 | 0.988 |
|  | 2020 | 103 | 73 |  |  | 8 |  |
|  |  |  |  |  |  |  |  |
| Mixed dishes | 2017 | 55 | 91 | 0.141 |  | 5 | 0.025 |
|  | 2020 | 90 | 97 |  |  | 0 |  |
|  |  |  |  |  |  |  |  |
| Savoury snacks | 2017 | 12 | 67 | 0.049 |  | 0 | n/a |
|  | 2020 | 40 | 90 |  |  | 0 |  |
|  |  |  |  |  |  |  |  |
| Salted Nut Butters^‡^ | 2010 | 30 | 43 | 0.262 |  | 17 | 0.518 |
|  | 2013 | 35 | 29^d^ |  |  | 29^d^ |  |
|  | 2017 | 51 | 57^df^ |  |  | 10^d^ |  |
|  | 2020 | 78 | 26^f^ |  |  | 18 |  |

**Note:** Pairwise comparisons between proportions for each year-pair (i.e., 2010-2013, 2010-2017, 2010-2020, 2013-2017, 2013-2020, and 2017-2020) were made by contrasting estimated marginal means from Firth's bias-reduced logistic regression model. p-trend was calculated using Cochran-Armitage tests with significance level p<0.05. Analysis by major category only is provided in Table 2 and overall in Figure 2.

^*^Health Canada’s 2012-2016 voluntary sodium reduction targets were published in the *Guidance for the Food Industry on Reducing Sodium in Processed Foods* document. Available at: <https://www.canada.ca/en/health-canada/services/food-nutrition/legislation-guidelines/guidance-documents/guidance-food-industry-reducing-sodium-processed-foods-2012.html>.

^†^Models were not fit for pairwise comparisons with n<10 in either year. n/a, p-trend was not calculated for categories with <10 products in any year.

^‡^There is only one subcategory under the canned and bottled vegetables and legumes and salted nut butters major categories.

^§^Infant and Toddler Foods were not collected in the FLIP 2010 and 2013 collections.

^a^p<0.05 for comparison between 2010-2013 from logistic regression.

^b^p<0.05 for comparison between 2010-2017 from logistic regression.

^c^p<0.05 for comparison between 2010-2020 from logistic regression.

^d^p<0.05 for comparison between 2013-2017 from logistic regression.

^e^p<0.05 for comparison between 2013-2020 from quantile regression.

^f^p<0.05 for comparison between 2017-2020 from quantile regression.


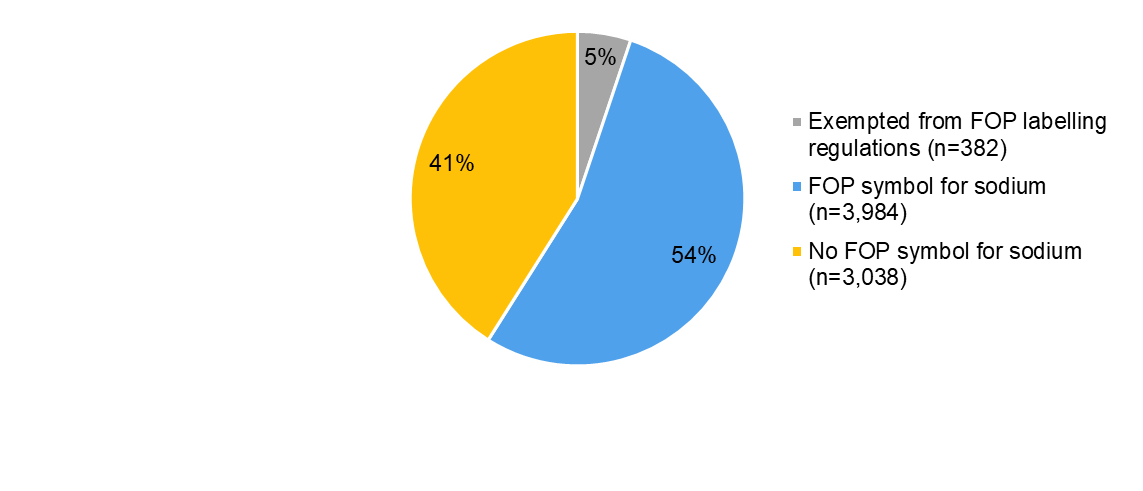


**Supplementary Figure 1.** The proportion of products in FLIP 2020 that exceeded the 2016 average sodium reduction targets^*^ that would be required to carry a front-of-package (FOP) “High in” symbol for sodium under Health Canada’s FOP labelling regulations^†^. Due to missing data required to evaluate products against the FOP labelling regulations, 2,826 of the 10,230 products that exceeded Health Canada’s average sodium reduction targets were excluded.

^*^Health Canada’s 2012-2016 voluntary sodium reduction targets were published in the *Guidance for the Food Industry on Reducing Sodium in Processed Foods* document. Available at: <https://www.canada.ca/en/health-canada/services/food-nutrition/legislation-guidelines/guidance-documents/guidance-food-industry-reducing-sodium-processed-foods-2012.html>.

^†^FOP labelling regulations were applied as published in Canada Gazette Part II in 2022, including the expanded exemption eligibility for diary-related products that was published in the 2024 Marketing Authorization. Canada Gazette Part II is available at: <https://canadagazette.gc.ca/rp-pr/p2/2022/2022-07-20/html/sor-dors168-eng.html>. The Marketing Authorization is available at: <https://gazette.gc.ca/rp-pr/p2/2024/2024-06-05/html/sor-dors89-eng.html>.
